# African savanna raptors show evidence of widespread population collapse and a growing dependence on protected areas

**DOI:** 10.1038/s41559-023-02236-0

**Published:** 2024-01-04

**Authors:** Phil Shaw, Darcy Ogada, Leah Dunn, Ralph Buij, Arjun Amar, Rebecca Garbett, Marc Herremans, Munir Z. Virani, Corinne J. Kendall, Barbara M. Croes, Martin Odino, Shiv Kapila, Peter Wairasho, Christian Rutz, André Botha, Umberto Gallo-Orsi, Campbell Murn, Glyn Maude, Simon Thomsett

**Affiliations:** 1 Centre for Biological Diversity, School of Biology, University of St Andrews, St Andrews, UK; 2 The Peregrine Fund, Boise, ID USA; 3 National Museums of Kenya, Nairobi, Kenya; 4 Animal Ecology Group, Wageningen University and Research, Wageningen, the Netherlands; 5 FitzPatrick Institute of African Ornithology, DST-NRF Centre of Excellence, University of Cape Town, Cape Town, South Africa; 6 Southern Africa Leopard Project, Panthera, Cape Town, Western Cape South Africa; 7 Natuurpunt Studie vzw, Mechelen, Belgium; 8 Mohamed Bin Zayed Raptor Conservation Fund, Abu Dhabi, United Arab Emirates; 9 North Carolina Zoo, Asheboro, NC USA; 10 Department of Applied Ecology, North Carolina State University, Raleigh, NC USA; 11 Institute of Environmental Sciences, Leiden University, Leiden, the Netherlands; 12 The Kenya Bird of Prey Trust, Naivasha, Kenya; 13 Endangered Wildlife Trust, Gauteng, South Africa; 14 Raptors MOU Coordinating Unit, Convention on Migratory Species (CMS), Abu Dhabi, United Arab Emirates; 15 Hawk Conservancy Trust, Andover, Hampshire, UK; 16 School of Biological Sciences, University of Reading, Berkshire, UK; 17 Raptors Botswana, Maun, Botswana

**Keywords:** Conservation biology, Biodiversity

## Abstract

The conversion of natural habitats to farmland is a major cause of biodiversity loss and poses the greatest extinction risk to birds worldwide. Tropical raptors are of particular concern, being relatively slow-breeding apex predators and scavengers, whose disappearance can trigger extensive cascading effects. Many of Africa’s raptors are at considerable risk from habitat conversion, prey-base depletion and persecution, driven principally by human population expansion. Here we describe multiregional trends among 42 African raptor species, 88% of which have declined over a ca. 20–40-yr period, with 69% exceeding the International Union for Conservation of Nature criteria classifying species at risk of extinction. Large raptors had experienced significantly steeper declines than smaller species, and this disparity was more pronounced on unprotected land. Declines were greater in West Africa than elsewhere, and more than twice as severe outside of protected areas (PAs) than within. Worryingly, species suffering the steepest declines had become significantly more dependent on PAs, demonstrating the importance of expanding conservation areas to cover 30% of land by 2030—a key target agreed at the UN Convention on Biological Diversity COP15. Our findings also highlight the significance of a recent African-led proposal to strengthen PA management—initiatives considered fundamental to safeguarding global biodiversity, ecosystem functioning and climate resilience.

## Abstract (web summary)

A compilation of survey data from pre- and post-2000 for 42 raptor species across parts of West, Central, East and southern Africa shows 88% of species in population decline and reveals trends across regions, protected areas and species size.

## Main

The conversion of wooded habitats to agricultural land is more damaging to biodiversity than any other human activity^1–4^ and poses the greatest extinction risk to birds worldwide^2,3^. Tropical raptors are especially vulnerable, being particularly slow-breeding^5,6^ and subject to a wide range of threats linked to rapid human population growth, farmland expansion^7–10^ and habitat fragmentation^11^. While resident tropical raptors thus have great potential as a model system for investigating land-use change impacts, trends in their abundance have been little studied so far, reflecting the paucity of suitable long-term survey data and a limited capacity for conservation research in most developing countries^12^. Here we present a multiregional assessment of trends among many of Africa’s widespread, diurnal raptor species, and compare rates of change in their abundance within protected and unprotected areas.

Africa is exceptionally important for global raptor conservation, supporting high numbers of threatened species^13^. Over the past ca. 60 yr, however, the continent’s human population has expanded rapidly^10^, driving widespread land conversion and habitat degradation, and creating areas where cumulative human impacts on threatened raptors are especially acute^9^. Sub-Saharan Africa lost almost 5 million ha of forest and non-forest natural vegetation per annum during 1975–2000 alone^14^ and now experiences the most severe rate of land degradation in the world^15^. With its human population projected to double by 2058, demands for grazing, arable land and energy are expected to rise substantially^10,16^. These trends will amplify existing pressures on Africa’s protected areas (PAs), which currently account for just 14% of its land and inland waters^17^. Although many PAs are considered to be failing or deteriorating^18,19^, well-managed sites form a critical refuge for the continent’s declining raptor populations^20–23^.

Additional threats to Africa’s avian apex predators, meso-predators and scavengers include prey-base depletion^13^, persecution (shooting, trapping, poisoning)^24^, unintentional poisoning^25^, electrocution/collision with energy infrastructure^26–29^ and killing for food and belief-based uses^30–32^. These pressures are typically more acute within unprotected land and have probably impacted larger raptor species more severely, reflecting global patterns of extinction risk among terrestrial mammalian predators^33^. Importantly, the loss and depletion of predator populations not only affects the species concerned, but can also trigger extensive cascading effects among their prey populations, disrupting ecosystem functioning^9,34–37^. Ecosystem services provided by raptors include the rapid removal of carcasses, potentially limiting the transmission of zoonotic diseases to human populations^37–39^.

**Fig. 1:**
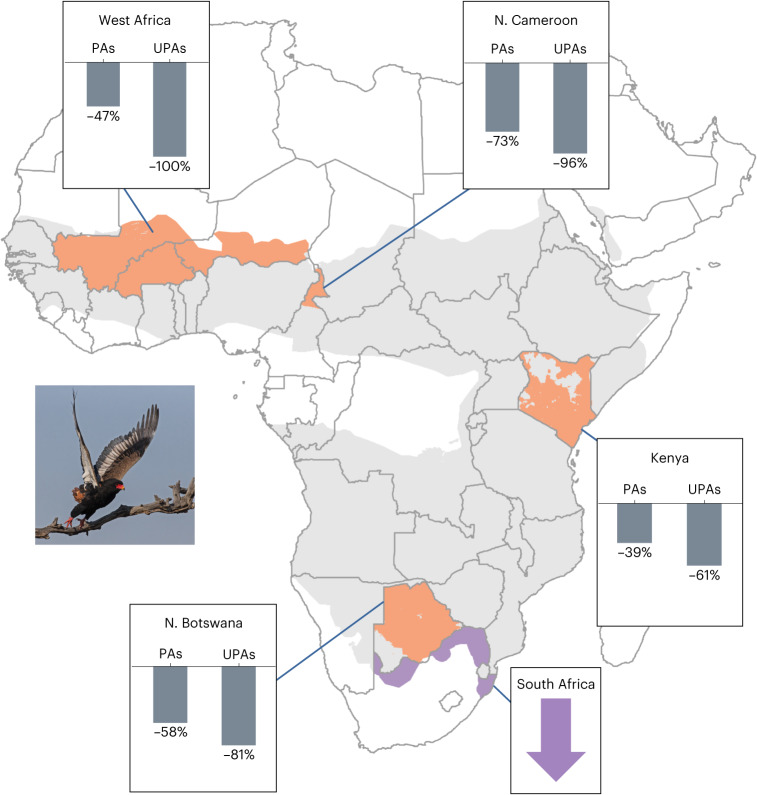
Trend estimates were derived from four road transect studies and a bird atlas project, located in West, Central, East and southern Africa. Road transects were conducted in West Africa, northern Cameroon and Kenya in 1969–1977 and 2000–2020, and in northern Botswana in 1991–1995 and 2015–2016. Here, orange shading indicates parts of the global range of bateleur *Terathopius ecaudatus* that lie within road transect countries and overlap with areas where climatic conditions match those of the routes surveyed in that country. Grey shading indicates the rest of the species’ range within surveyed and unsurveyed countries alike. Bar charts show percentage change in the number of individuals encountered per 100 km within protected and unprotected areas (PAs and UPAs), projected over three generation lengths; 44 yr in this instance. The species’ trajectory within its South African range (mauve) was derived from SABAP2 reporting rates during 2008–2021. Photograph: © André Botha. Source data

**Extended Data Fig. 1:**
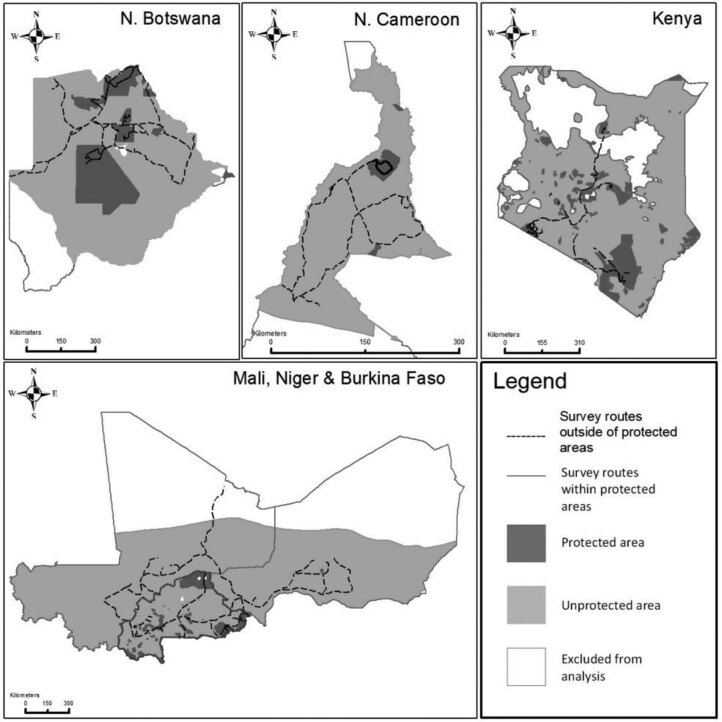
Routes surveyed during four road transect studies. These were conducted during 1969–1977 and 2000–2020 in northern Cameroon, Kenya, Burkina Faso, Niger and Mali, and during 1991–1995 and 2015–2016 in northern Botswana. Panels adapted with permission from: Burkina Faso, Niger and Mali, ref. ^40^, Wiley; northern Cameroon, ref. ^41^, Allen Press; Kenya, ref. ^42^, Elsevier; northern Botswana, ref. ^43^, Elsevier.

Despite these pressures, and the keystone role played by many raptor species, attempts to measure trends in their abundance have been hindered by the absence of systematic, pan-African bird monitoring programmes, generating robust, long-term trend data for this species group. Here, based on repeated raptor road transect surveys undertaken in four African regions, we examine changes in encounter rates (individuals recorded per 100 km) among 42 species dependent mainly on savanna habitats. To determine rates of change, we combined published and unpublished road transect data from surveys conducted during 1969–1995 and 2000–2020 in West Africa (Burkina Faso, Niger and Mali)^40^, Central Africa (northern Cameroon)^41^, East Africa (Kenya)^42^ and southern Africa (northern Botswana)^20,43^ (Fig. 1, Supplementary Table 1 and Extended Data Fig. 1). Pooling these data has provided unprecedented insights into trends in the abundance of Africa’s savanna raptors, enabling us to identify species whose composite decline estimates exceed the limits defining their current International Union for Conservation of Nature (IUCN) threat status. We also determine the extent to which decline rates differed between selected PA categories and unprotected land, and investigate potential links between abundance change, body size and protected area dependency.

## Results

**Fig. 2:**
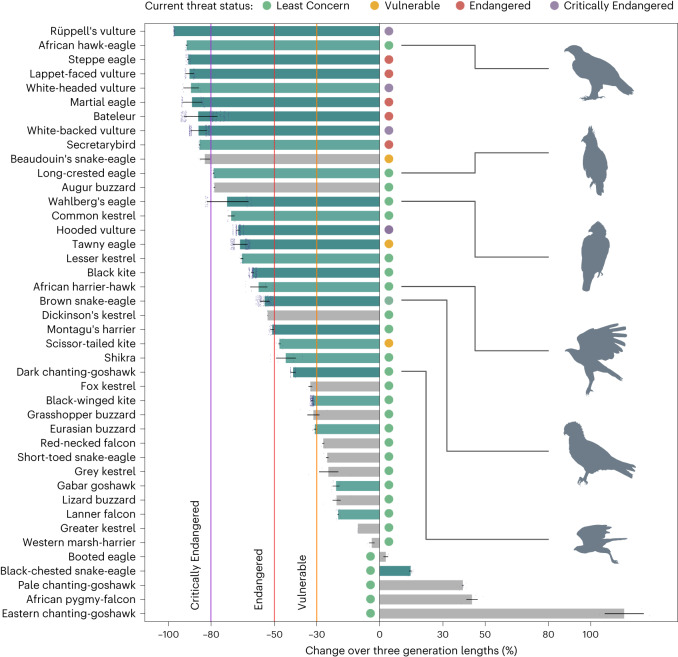
Percentage change in the number of individuals encountered per 100 km during road transect surveys, projected over three generation lengths. Fifteen species were surveyed adequately in single regions only (grey bars). The remaining 27 were each surveyed in two regions (lighter green bars) or 3–4 regions (dark green). Bar length shows a given species’ median rate of change in abundance, estimated under two scenarios, in which average encounter rates in unsurveyed PAs were assumed to have been the same as in surveyed PAs, or the same as in UPAs (Methods). Points overlaid on bars show individual change estimates, where the sample size (*n* = 4, 16, 64 or 256) reflects the number of studies in which the species was surveyed (1, 2, 3 or 4 studies); error bars show the Q1–Q3 range. Twenty-nine species had declined at rates exceeding the IUCN Vulnerable threshold; 24 had exceeded the limits defining their current threat category. Fifteen of these are African endemics or near-endemics, 6 of which (illustrated) were surveyed in multiple regions and are currently listed as Least Concern. Silhouettes drawn from photographs: © André Botha. Source data

We found strong evidence of widespread declines among African raptor species spanning up to 40 yr (Table 1). Overall, 37 (88%) of the 42 species examined had declined, 29 (69%) by at least 30% over three generation lengths—a criterion used by IUCN to identify species at risk of global extinction^44^. Of 27 species surveyed in multiple regions, 24 (89%) had exceeded this decline threshold (Fig. 2), 13 of which are currently classified as Least Concern^45^. While 7 of these 13 species have extensive global ranges outside of Africa, where trends may differ from those reported here, the remaining 6 are African endemics or near-endemics (Fig. 2).

**Table 1:** Changes in the number of individuals encountered per 100 km during road transect studies

Species^a^	Migratory status^b^	Current IUCN status^c^	Change over three generation lengths (%)^d^						
			Median	Quartiles	West Africa	Northern Cameroon	Kenya	Northern Botswana	South Africa^e^
**Secretarybird** ***Sagittarius serpentarius***	AS	EN	−85	−85.1 −85.7	−	−	−89	−82	**⇩**
Black-winged kite *Elanus caeruleus*	AS	LC	−32	−31.7 −32.6	−18	+74	−36	−58	**⇩**
Scissor-tailed kite *Chelictinia riocourii*	AM	VU	−48	−47.1 −48.1	−48	+24	−	−	−
Black kite *Milvus migrans*	PMAM	LC	−60	−59.6 −61.0	−64	−41	−40	−64	⇩
Hooded vulture *Necrosyrtes monachus*	AS	CR	−67	−66.2 −68.0	−51	−51	−88	−80	⇧
White-backed vulture *Gyps africanus*	AS	CR	−86	−81.8 −89.6	−95	−71	−74	−20	⇩
Rüppell’s vulture *Gyps rueppelli*	AS	CR	−97	−97.3 −97.6	−98	−88	−21	−	−
**Lappet-faced vulture** ***Torgos tracheliotos***	AS	EN	−90	−88.0 −92.0	−97	−	−69	−76	⇩
White-headed vulture *Trigonoceps occipitalis*	AS	CR	−90	−85.6 −93.0	−94	−	−	−77	**⇩**
Short-toed snake-eagle *Circaetus gallicus*	PM	LC	−25	−24.4 −25.6	−	−25	−	−	−
**Beaudouin’s snake-eagle** ***Circaetus beaudouini***	AM	VU	−83	−80.4 −85.3	−83	−	−	−	−
Black-chested snake-eagle *Circaetus pectoralis*	AS	LC	+15	+14.3 +15.6	−	−	−29	+77	⇧
**Brown snake-eagle** ***Circaetus cinereus***	AS	LC	−55	−52.3 −56.9	−78	−71	−15	+67	⇧
**Bateleur** ***Terathopius ecaudatus***	AS	EN	−87	−76.9 −92.8	−91	−89	−50	−75	**⇩**
Western marsh-harrier *Circus aeruginosus*	PM	LC	−4	−2.6 −5.3	−4	−	−	−	−
Montagu’s harrier *Circus pygargus*	PM	LC	−51	−50.2 −51.9	−50	+12	−59	−	**⇩**
**African harrier-hawk** ***Polyboroides typus***	AS	LC	−58	−53.3 −61.5	−64	−	−	−34	**⇧**
**Dark chanting-goshawk** ***Melierax metabates***	AS	LC	−41	−40.0 −42.3	−44	−41	−	−23	**⇩**
Eastern chanting-goshawk *Melierax poliopterus*	AS	LC	+116	+106.8 +125.3	−	−	+ 116	−	−
Pale chanting-goshawk *Melierax canorus*	AS	LC	+40	+39.3 +40.2	−	−	−	+ 40	**⇩**
Gabar goshawk *Micronisus gabar*	AS	LC	−21	−19.3 −22.5	−23	−	−	−14	**⇧**
Lizard buzzard *Kaupifalco monogrammicus*	AS	LC	−21	−18.7 −22.6	−21	−	−	−	**⇩**
Shikra *Accipiter badius*	AM	LC	−45	−39.8 −49.3	−32	−	−	−65	**⇩**
**Grasshopper buzzard** ***Butastur rufipennis***	AM	LC	−32	−28.7 −34.4	−32	−	−	−	−
Eurasian buzzard *Buteo buteo*	PM	LC	−31	−30.3 −31.4	−	−	+ 36	−54	**⇩**
**Augur buzzard** ***Buteo augur***	AS	LC	−78	−78.0 −78.7	−	−	−78	−	−
Tawny eagle *Aquila rapax*	AS	VU	−66	−62.7 −69.6	−91	−71	−7	+93	**⇧**
Steppe eagle *Aquila nipalensis*	PM	EN	−91	−90.2 −91.3	−	−56	−78	−96	**⇩**
**African hawk-eagle** ***Aquila spilogaster***	AS	LC	−91	−91.1 −91.8	−84	−	−	−97	**⇧**
**Wahlberg’s eagle** ***Hieraaetus wahlbergi***	AM	LC	−74	−62.2 −81.9	−81	−	−32	−48	⇧
Booted eagle *Hieraaetus pennatus*	PMAM	LC	+3	+2.1 +4.4	+3	−	−	−	**⇧**
**Martial eagle** ***Polemaetus bellicosus***	AS	EN	−90	−84.0 −93.6	−97	−	−23	−56	⇩
**Long-crested eagle** ***Lophaetus occipitalis***	AS	LC	−79	−78.4 −79.1	−	−66	−80	−	**⇧**
African pygmy-falcon *Polihierax semitorquatus*	AS	LC	+44	+41.3 +46.7	−	−	+ 44	−	**⇩**
Lesser kestrel *Falco naumanni*	PM	LC	−65	−64.7 −66.0	−	−	−53	−74	**⇩**
Common kestrel *Falco tinnunculus*	PMAS	LC	−70	−68.8 −72.0	−	−65	−71	−	**⇩**
Greater kestrel *Falco rupicoloides*	AS	LC	−11	−10.5 −10.6	−	−	−	−11	**⇩**
**Fox kestrel** ***Falco alopex***	AS	LC	−33	−32.1 −33.9	−33	−	−	−	−
Grey kestrel *Falco ardosiaceus*	AS	LC	−25	−19.8 −29.0	−25	−	−	−	−
**Dickinson’s kestrel** ***Falco dickinsoni***	AS	LC	−53	−53.1 −53.3	−	−	−	−53	−
Red-necked falcon *Falco ruficollis*	AS	LC	−27	−26.4 −27.5	−27	−	−	−	−
Lanner falcon *Falco biarmicus*	AS	LC	−20	−19.6 −20.2	−19	−	−	−22	**⇧**

Encounter rate changes for each species were estimated from studies conducted in West Africa, northern Cameroon and Kenya (1969–1977 to 2000–2020) and in northern Botswana (1991–1995 to 2015–2016). These were annualized, averaged across studies (weighted by the species’ range size in each study area) and projected over three generation lengths. Fifteen species shown in bold are African endemics or near-endemics whose decline estimates exceed the limits defining their current IUCN threat status. Trends among 30 of the 42 species were also determined in South Africa, from SABAP2 reporting rates recorded during 2008–2021; bold arrows indicate *P* < 0.05.

^a^Species are listed in taxonomic order, following ref. ^52^.

^b^Migratory status: AS, Afrotropical sedentary; AM, Afrotropical migrant; PM, Palaearctic migrant. Sources: refs. ^21,52^.

^c^IUCN global threat status: LC, Least Concern; VU, Vulnerable; EN, Endangered; CR, Critically Endangered^45^.

^d^Median, Q1 and Q3 rates of change over three generation lengths were derived from two scenarios, in which average encounter rates in unsurveyed PAs were assumed to have either been the same as in surveyed PAs, or the same as in UPAs, respectively (Methods).

^e^Species meeting data selection criteria in fewer than 30 SABAP2 pentads (‘−’) were excluded from the analysis (Methods).

### Large raptors showed more rapid declines

**Fig. 3:**
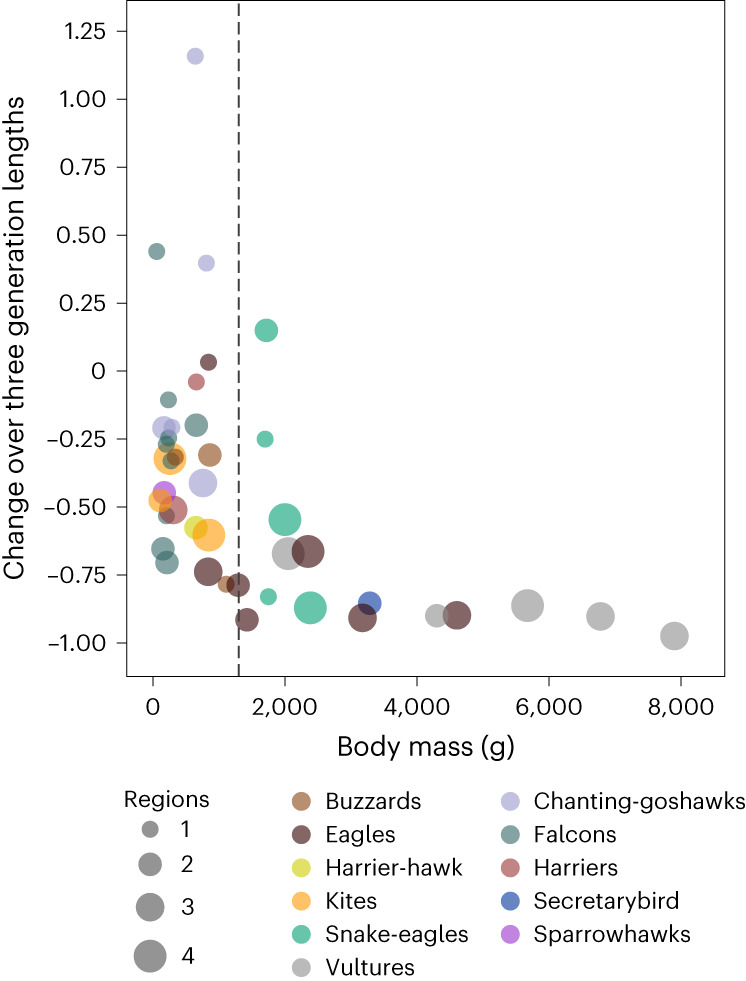
Encounter rate changes projected over three generation lengths in relation to body mass. Each point represents one species (*n* = 42), grouped taxonomically as in Supplementary Table 2. Circle size indicates the number of regions in which the species was surveyed (*n* = 1–4). Rates of change were more variable among small–medium raptors than among large species (≥1,300 g; dashed line). Most large raptors had declined by at least 80% over three generation lengths, partly reflecting the positive relationship between body mass and longevity. Source data

The annual rate of change in encounter rates within the four regions combined was inversely related to body mass, with larger species showing significantly steeper declines (effect size = –0.016 × sqrt(mass, kg), *R*^2^ = 0.109, *P* = 0.0185; model 1 in Extended Data Table 1). This relationship was amplified when projected over three generation lengths (effect size = –0.351 × sqrt(mass, kg), *R*^2^ = 0.254, *P* = 0.0004; model 2 in Extended Data Table 1), since generation length itself is positively correlated with body mass (effect size = 2.555 × log(mass, g), *R*^2^ = 0.830, *P* < 0.0001; model 3 in Extended Data Table 1). We note, however, that this pattern was strongly influenced by the 10 heaviest species, all of which had declined at rates exceeding 60% over three generation lengths (Fig. 3). Thus, larger, apex raptors and scavengers had declined more rapidly per annum than smaller species, and since larger species tend to live longer, this relationship was more pronounced when projected over three generation lengths.

### Rates of change varied between regions

Raptor population decline rates were significantly more severe in West Africa than elsewhere. In Central, East and southern Africa, there was no significant regional variation in encounter rate trends (*χ*^2^_2_ = 0.2113, *P* = 0.8997; model 4 in Extended Data Table 1), and the median annual rate of change was −2.3%. In West Africa, encounter rates for the same species had declined more than twice as rapidly, at a median of −5.4% per annum (*χ*^2^_1_ = 13.288, *P* = 0.0003; model 5 in Extended Data Table 1).

To extend our geographical coverage within southern Africa, we determined the direction of change in atlas reporting rates in South Africa for 30 of the 42 species, using data from the Southern African Bird Atlas Project (SABAP2) spanning 2008–2021^46^. Reporting rates for 15 species had changed significantly (Bonferroni correction applied), of which 9 (60%) had suffered declines. Ten of the 15 species showed the same direction of change in South Africa as was evident from road transect surveys elsewhere (Table 1; concordance no greater than chance: *χ*^2^_1_ = 1.666, *P* = 0.1967).

Decline rates derived from road transect surveys showed a negative but non-significant association with migratory status, after controlling for body-mass effects. The mean annual rate of decline among 14 species that are either migratory or have both migratory and sedentary populations in Africa was 52% higher than among 28 wholly sedentary species (effect size = 0.015, *R*^2^ = 0.170, *P* = 0.0989; model 6 in Extended Data Table 1).

### Raptor declines were less severe within PAs than elsewhere

**Fig. 4:**
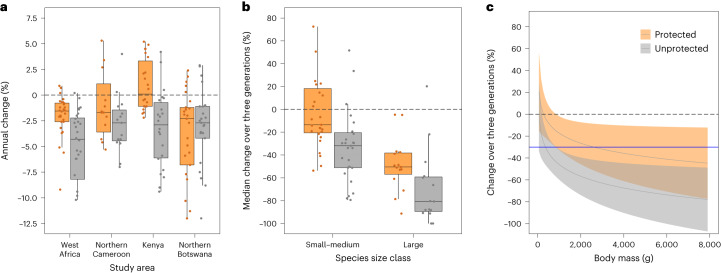
The effects of protected area status and body mass on rates of change. Results from protected and unprotected areas are shown in orange and grey, respectively. **a**, In all four road transect studies, median annual decline rates in UPAs exceeded those within the PAs assessed, significantly so in West Africa and Kenya. Boxplots show the median, first and third quartiles of the change in abundance within protected and unprotected areas in each of the four regions. Whiskers extend to ±1.5× the interquartile range. Each point represents one species; *n* = 28 (West Africa), 15 (N. Cameroon), 22 (Kenya) and 25 (N. Botswana). **b**, The effects of site protection were more pronounced among large (≥1,300 g) than among small–medium raptors. Median decline rates in PAs vs UPAs differed by 30 percentage points among large raptors and by 18 percentage points among small–medium species. Boxplots show the median, first and third quartiles of the rate of change in abundance of large vs small–medium raptor species, inside vs outside of protected areas. Whiskers extend to ±1.5× the interquartile range. Each point represents one species; *n* = 15 large, 27 small–medium species. **c**, Modelled relationship between the rate of change in abundance, body mass and protected area status (PAs vs UPAs). Notably, declines over three generation lengths exceeded the IUCN Vulnerable threshold (−30%, blue line) for the bulk of species in UPAs and for most large raptors within the PA types assessed. Fitted lines and shading indicate modelled change rates ±1 s.e.m. (model 13 in Extended Data Table 1). Source data

In each region, the median annual decline rate was greater in unprotected areas (UPAs) than within the protected area types assessed here (Fig. 4a), significantly so in the case of West Africa (Wilcoxon signed-ranks test: *V* = 349, *P* = 0.0005; model 7 in Extended Data Table 1) and Kenya (*V* = 229, *P* = 0.0004; model 8 in Extended Data Table 1). Overall, 33 (79%) of the 42 species had declined more rapidly in UPAs, as had 24 (89%) of the 27 species surveyed in multiple regions. The median annual rate of decline among the 42 species assessed was 2.3 times higher in UPAs (−2.66%, quartiles: −1.74% to −5.25%) than in PAs (−1.15%, quartiles: +0.06% to −2.18%) (*V* = 792, *P* < 0.0001; model 9 in Extended Data Table 1). Similarly, the median rate of decline over three generation lengths was 2.5 times higher in UPAs (−48%, quartiles: −27% to −78%) than in PAs (−19%, quartiles: +1% to −49%) (*V* = 765, *P* < 0.0001; model 10 in Extended Data Table 1). Thus, while many species had declined in both protected and unprotected areas, annual rates of decline were more than twice as high in the latter.

When PA effects were controlled for, large raptors (>1,300 g; Supplementary Table 2) continued to show steeper annual declines than smaller species (*χ*^2^_1_ = 5.781, *P* = 0.0162; model 11 in Extended Data Table 1). Projected over three generation lengths, decline rates of large raptors were substantially higher than those of smaller species, within PAs (median change: −50.5% vs −13.5%) as well as UPAs (−80.7% vs −31.9%) (*χ*^2^_1_ = 20.942, *P* < 0.0001; model 12 in Extended Data Table 1). The influence of body mass on decline rate was thus greater on unprotected land (a difference of 49 percentage points) than on protected land (37 percentage points) (*χ*^2^_1_ = 10.491, *P* = 0.0012; model 12 in Extended Data Table 1) (Fig. 4b). Notably, even within PAs, decline rates of most large species had exceeded the IUCN Vulnerable threshold (−30% over three generation lengths) (Fig. 4c; model 13 in Extended Data Table 1). Indeed, 17 (40%) of the 42 species had declined within PAs at rates exceeding the Vulnerable, Endangered or Critically Endangered threshold, compared with 27 species (64%) in UPAs. Thus, although population declines within the PA types assessed were lower than elsewhere, particularly for large raptor species, in some cases they still exceeded IUCN thresholds classifying species at risk of extinction.

### Reliance on protected areas had increased significantly

**Fig. 5:**
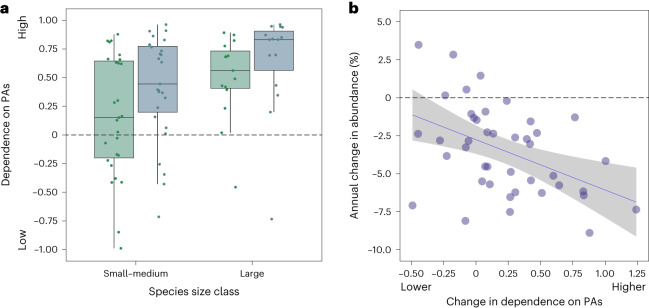
The number of individuals encountered per 100 km in protected versus unprotected areas, as an index of each species’ dependence on PAs. Index values potentially ranged from +1.0 (recorded only within PAs) to −1.0 (recorded only in UPAs). **a**, Boxplot showing PA dependency scores in relation to survey period (green, 1969–1995; blue, 2000–2020) and body size class. In each period, large raptors were significantly more dependent on PAs than small–medium species. Notably, for species in both size classes, PA dependency increased significantly between 1969–1995 and 2000–2020. Boxplots show the median, first and third quartiles. Whiskers extend to ±1.5× the interquartile range. Each point represents one species; *n* = 15 large, 27 small–medium. **b**, Scatterplot showing annual change in abundance vs change in dependency on protected areas.The extent to which a species’ dependence on PAs changed between the two periods was significantly correlated with change in abundance. Species whose encounter rates had declined sharply had become more dependent on PAs than those showing a moderate decline or increase. Each point represents one species (*n* = 42); the fitted line and shading show modelled change rates ±1 s.e.m. (model 16 in Extended Data Table 1). Source data

To further examine the role of protected areas as potential refugia for raptor populations, we measured the disparity between each species’ encounter rates within the PA types we assessed and in UPAs, as an index of its dependence on the former. A positive index value indicated a higher encounter rate within PAs, and values potentially ranged from +1.0 (recorded only in PAs) to −1.0 (recorded only in UPAs). In each survey period, large raptors were significantly more dependent on PAs than were smaller species (*χ*^2^_1_ = 4.461, *P* = 0.0346, *n* = 84; model 14 in Extended Data Table 1). Between the two periods, 29 (69%) of the 42 species had become more dependent on PAs, with the median dependency score rising from 0.56 to 0.83 for large raptors and from 0.15 to 0.44 for smaller species (*χ*^2^_1_ = 12.151, *P* = 0.0005, *n* = 84; model 14 in Extended Data Table 1) (Fig. 5a).

The widening disparity between raptor abundance levels in PAs and UPAs was driven by differences in decline rates. While encounter rates in UPAs fell by a median of 54% (Wilcoxon signed-rank test: *V* = 849.0, *P* < 0.0001; model 15 in Extended Data Table 1), in PAs they fell by a median of 19% (*V* = 633.5, *P* < 0.0232; model 15 in Extended Data Table 1). Thus, raptors had become less abundant both within PAs and UPAs, indicating that the growing disparity arose more from a rapid deterioration in conditions outside of protected areas than from improving or stable conditions within.

### Rapidly declining species had become more PA-dependent

Interestingly, the rate of change in abundance was correlated with change in a species’ dependence on protected areas (effect size = −0.033, *R*^2^ = 0.189, *P* = 0.0024; model 16 in Extended Data Table 1). However, since both measures were derived from encounter rate values, we caution that the nature of this relationship may have been influenced by a high level of endogeneity within the model. Nevertheless, our findings indicate that species suffering the sharpest drop in abundance had become more dependent on protected areas than those showing little or no change (Fig. 5b).

## Discussion

Over periods of ca. 20–40 yr, many of the 42 African raptor species examined had endured a double jeopardy – of precipitous population declines coupled with an increasing reliance on protected areas. While declines on a similar geographic scale have been reported previously for African vultures^47^, this study encompasses a much larger, more ecologically diverse group of savanna predators and scavengers, whose trajectories are more likely to reflect the broad range of pressures now facing African raptor populations.

Our trend analyses leveraged published road transect studies, whose key findings were in broad agreement with those of single-species studies employing more tailored survey methods^22,48–50^. They indicate that as a group, Africa’s diurnal raptors are facing an extinction crisis, with more than two-thirds of the species examined potentially qualifying as globally threatened. Notably, 13 of those surveyed in multiple regions are currently listed by IUCN as Least Concern (Table 1). A further 6 species recognized as globally threatened (secretarybird *Sagittarius serpentarius*, lappet-faced vulture *Torgos tracheliotos*, bateleur, tawny eagle *Aquila rapax*, steppe eagle *A. nipalensis* and martial eagle *Polemaetus bellicosus*) had declined more rapidly than the threshold rates used to define their current threat status. Our findings thus highlight the need to reassess their status at the earliest opportunity.

In contrast, our decline rate for hooded vulture (−67% over three generations) was much lower than that estimated in 2016^47^ (−83%) and on which the species’ current threat status (Critically Endangered) was initially based. This follows a recent review^51^ in which the species’ generation length estimate was substantially shortened, reducing the apparent scale of its decline over three generation lengths. Hooded vulture remains Critically Endangered, however, following a surge in demand for vulture body parts in West Africa, its stronghold region^32,45^. Three additional species showing steep declines are augur buzzard *Buteo augur*, Dickinson’s kestrel *Falco dickinsoni* and Beaudouin’s snake-eagle *Circaetus beaudouini*. The latter is of particular concern, having declined by 80–85% over three generation lengths within a large (and probably representative) portion of its global breeding range^52^. The plight of these African endemics illustrates the pressing need for research into raptors with restricted breeding ranges.

We show that large African raptors have suffered steeper annual declines than smaller species, mirroring the pattern of extinction risk observed among terrestrial mammalian predators^33^. The risks to large-bodied species are compounded both by their biological traits (for example, low population density, delayed maturity and low annual fecundity^33,53^) and environmental factors (home ranges requiring extensive tracts of scarce, suitable habitat, thereby increasing the species’ exposure to human impacts). Furthermore, the loss of large-bodied species has a disproportionate effect on the resilience and functioning of ecosystems, as well as on human-centric values, such as revenue from tourism^54,55^.

### Declines were more pronounced in West Africa

Decline rates reported from West Africa^40,56,57^ were significantly more pronounced than those recorded elsewhere, consistent with the severity of threats documented in the region^31,32,40,56–58^, many being substantially worse there than elsewhere in sub-Saharan Africa^19,59^. Protected areas in West and Central Africa are particularly underfunded and mismanaged^19^, and high regional levels of poverty and corruption have been linked to adverse conservation outcomes for charismatic mammal species^59,60^. Furthermore, the rate of agricultural expansion in West Africa during the 1970s–2000s was more than three times that of Africa as a whole (Supplementary Information: Anthropogenic pressures). Hence, raptor declines seem likely to have continued in the region since road transect surveys were last conducted in the early 2000s, highlighting the need for repeat surveys. In contrast, SABAP2 reporting rates suggest that proportionately fewer species had declined in South Africa than elsewhere, albeit over a shorter, more recent timeframe (2008–2021).

Migrant species appear to have suffered steeper declines than residents, although this effect was statistically non-significant. Similarly, there was no significant relationship between the direction of change evident among Palaearctic migrants in Africa and in Europe^61^, perhaps reflecting disparities between the populations surveyed, or shifts in the over-wintering distributions of some Palaearctic migrant species^62^.

### Decline rates were often high within protected areas

Raptors of all sizes lead an increasingly perilous existence in African savannas, where food supplies and breeding sites have been drastically reduced and persecution by humans is now widespread^40,42,56,57^. While annual declines on unprotected land were thus often substantially higher than within the PAs we assessed, there is now widespread acknowledgement that many African PAs are also losing their ecological integrity^18,63,64^, thereby depriving threatened species of effective refugia. Indeed, the scale of this deterioration has been assessed in a recent study^19^, which showed that over 82% of land encompassed within 516 African conservation areas was considered to be failing or deteriorating. Moreover, vulture and eagle species can range widely across protected area boundaries, exposing them to retaliatory and sentinel poisoning by pastoralists and poachers, respectively^65^, and to persecution by livestock farmers. Consequently, levels of attrition were high even within the PA types we assessed, where 40% of species had declined at rates exceeding the IUCN Vulnerable threshold. Clearly, the size, connectivity and/or management of these PAs has failed to safeguard such highly mobile species, reflecting concerns that many African PAs are too small to protect large raptors adequately^66^.

### Study limitations

While our sample accounted for 40% of Africa’s 106 diurnal raptor species^52^, their trajectories may not be representative of trends among the remaining species, many of which are forest dependent. Globally, tropical forest raptors are at greater risk of extinction than those associated with savannas^8^, perhaps especially so in Africa, where net forest loss during 2010–2020 exceeded that of all other continents^67^. Geographically, North Africa represents a further, notable gap in our coverage. Here, many of the same threats prevail as elsewhere in Africa, and the limited evidence available^27–29^ suggests that raptor population trends in the region may be similar to those south of the Sahara.

Differing trends within PAs and UPAs could result from factors other than site protection, including the possibility that land encompassed within PAs was initially more favourable for raptors than land left unprotected, as indicated by disparities between PA and UPA encounter rates during early survey periods (Supplementary Table 3). To investigate this possibility we re-examined survey data from northern Botswana, demonstrating that PA and UPA encounter rates within the same 1° × 1° grid cells were higher than those from grid cells where PAs were absent, suggesting that high PA encounter rates were due in part to more favourable initial conditions (Supplementary Information: Comparing protected and unprotected areas). However, separating the effects of site protection from other factors would require a more rigorous counterfactual study design^68^ involving a before–after control–intervention (BACI) approach^69^, or the careful matching of ecologically similar transects from PAs and UPAs^70^. The application of a counterfactual approach thus remains the ‘gold standard’ for future analyses of PA effects, and we recommend caution when interpreting PA–UPA disparities.

Shrub encroachment within savanna habitats since the 1980s could have adversely affected raptor detectability, potentially contributing to the disparities observed between early and recent encounter rates. Since vegetation structure in the vicinity of survey transects was not assessed, we were unable to test whether changes in woody cover had occurred along the routes surveyed. Although widespread changes in shrub encroachment have been reported^14,71^, their effects are likely to have been small in comparison with many of the declines reported here. Moreover, although shrub encroachment would seem less likely to impede the detection of large soaring raptors, these species had shown some of the steepest declines (Supplementary Information: Detectability).

### Mitigating raptor declines

While ongoing efforts to protect Africa’s charismatic megafauna, including elephants *Loxodonta* spp.^72^ and lions *Panthera leo*^19,57^, help safeguard critical raptor habitats, raptors have distinct management requirements differing from those of large mammals. These include the protection of nesting trees and cliffs, the global adoption of bio-pesticides for locust control^73^, more effective management of *Quelea* control operations, and an improved understanding of the corridors and habitats required by migrant raptors. Mitigation is urgently required to end the extensive mortality caused by powerlines and windfarms^26–29^, particularly along migratory flyways. Innovation is needed to reduce mortalities caused by lethal pole and turbine designs, and better enforcement of regulations is required to prevent energy infrastructure from being built within protected and sensitive areas^74^.

The future of Africa’s raptors also rests on (1) effective legislation for species protection, (2) enhanced management of PAs, particularly in relation to tree loss, disturbance at nest sites, poaching and poisoning, (3) tighter coordination between government and conservation stakeholders^13^ and (4) both improved law enforcement and innovative economic incentives to counter persecution^24^, sentinel poisoning^65^ and the harvesting of raptors for food and belief-based use^30–32^. Better coordination is also required between range states encompassing migratory routes^75^, facilitated by frameworks such as the Convention on Migratory Species (CMS) Memorandum of Understanding (MOU) on the conservation of birds of prey in Africa and Eurasia.

To address the need for long-term raptor monitoring and expanded research and conservation programmes, we have developed the African Raptor Leadership Grant, which supports educational and mentoring opportunities, boosting local conservation initiatives and knowledge of raptors across the continent. Furthermore, we recommend increased stakeholder engagement in raptor conservation to develop regional raptor Red Lists, monitoring schemes and species action plans, with guidance from the CMS Raptor MOU Technical Advisory Group and relevant IUCN Species Specialist Groups.

The evidence we present here of a significant shift in the reliance of African raptor species on protected areas substantiates recent calls to expand the global protected area network^76,77^ and demonstrates the importance of proposals agreed at the Convention on Biological Diversity COP15 in 2022: to effectively conserve and manage at least 30% of the world’s surface by 2030^78^. Furthermore, our results underscore the need to substantially improve PA management throughout Africa, to meet the ‘green list standard’ set by the IUCN World Commission on Protected Areas^79^. In this regard, a recent African-driven initiative—APACT—may prove pivotal in leveraging the finances needed to effectively manage new and existing conserved areas^63^.

While raptors also extensively utilize unprotected areas, particularly during migration^80^ and seasonal stays^81^, human population projections for sub-Saharan Africa^10^ point to further, widespread conversion and degradation of natural habitats, particularly on unprotected land. Well-established links between land conversion and biodiversity loss^1–4,9,11^, together with the patterns of decline documented here, give cause to doubt whether large raptors will persist over much of Africa’s unprotected land in the latter half of this century. Broad-scale interventions and collaborations are thus urgently required to address the multitude of threats facing raptors in unprotected areas, thereby also helping to protect other wildlife species. Furthermore, there is a pressing need to substantially improve the connectivity, management and coverage of PAs in Africa, in line with global aspirations^77–79^—a transition considered fundamental to safeguarding biodiversity, ecosystem functioning and climate resilience^76^.

## Methods

### Road transect studies

We collated published results from road transect studies conducted in Burkina Faso, Niger and Mali (West Africa)^40^, Kenya (East Africa)^42,82^ and northern Botswana (southern Africa)^20,43^, together with published and unpublished survey results from northern Cameroon (Central Africa)^41^ (R.B. and B.M.C, unpublished data). These studies covered a combined survey distance of 94,151 km (Supplementary Table 1), yielding 53,209 sightings of the 42 study species. In each study, routes were surveyed during an ‘early’ and ‘recent’ period, separated by an interval of ca. 20–40 yr. For each raptor species in each study and survey period, we calculated an average encounter rate (individuals seen per 100 km) separately for routes lying within PAs and UPAs (Extended Data Fig. 1). Protected areas were defined by the authors of the original studies, who excluded site categories affording little or no meaningful protection for wildlife, or where the degree of protection provided was uncertain (Supplementary Information: Survey routes and protected areas). In the absence of historical digital maps, contemporary PA boundaries^17^ were used when estimating land areas during early and recent survey periods. Where insufficient detail had been provided, PA types were confirmed subsequently by the lead author of the study in question (Supplementary Table 4). To minimize chance effects, we restricted our analyses to species for which at least 20 individuals had been recorded in a given study area during the early survey period, with at least five sightings each in PAs and UPAs. Potential effects of excluding cases with smaller sample sizes are considered in Supplementary Information: Case selection.

### Estimating change in encounter rates

**Extended Data Fig. 2:**
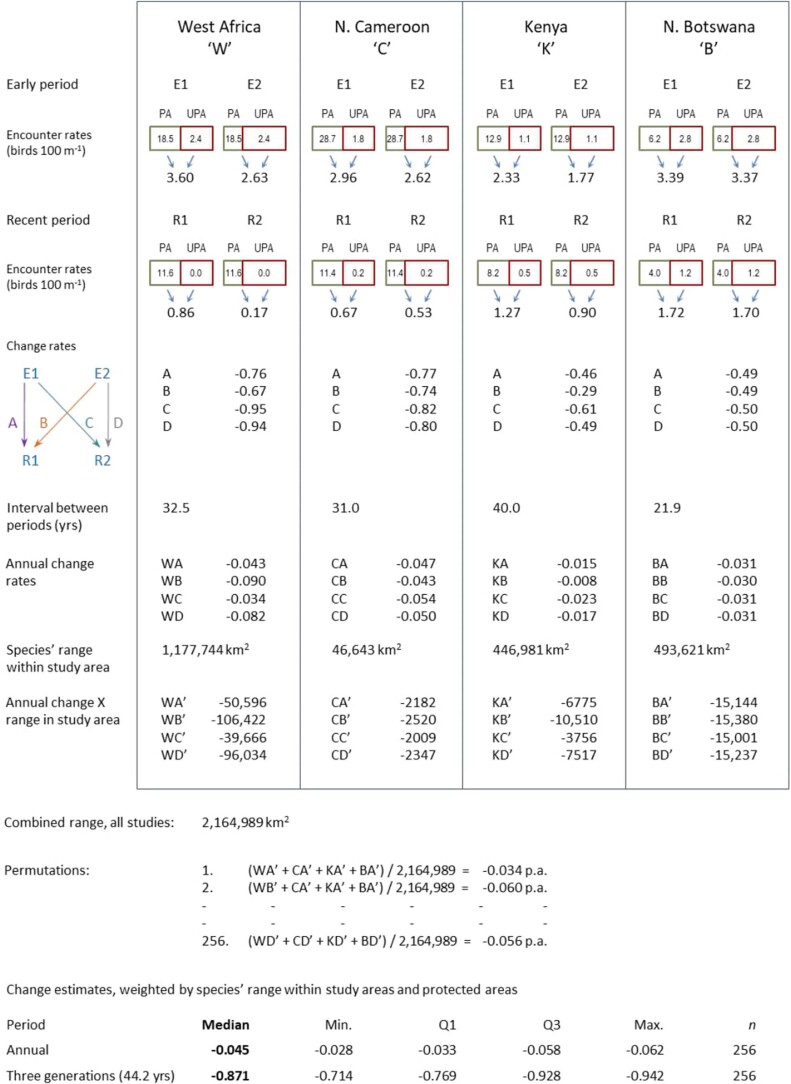
Method used to produce a composite estimate of the rate of change in abundance. The survey data used here are for bateleur *Terathopius ecaudatus*, and were drawn from all four road transect studies. Average encounter rates within PAs and UPAs are shown for early (E) and recent (R) survey periods. For each period, we combined these to produce a weighted average for the study area in question, based on two scenarios, in which the average encounter rate within unsurveyed PAs was assumed either to be (1.) the same as in surveyed PAs, or (2.) the same as in UPAs. The land area to which the PA encounter rate was assumed to apply thus differed between these two scenarios, as indicated by the relative sizes of the green (PA) and red (UPA) boxes shown, exaggerated here for illustrative effect. These encounter rate values yielded four estimates of change for each study area between survey periods, corresponding to E1– > R1, E1– > R2, E2– > R1 and E2– > R2, as illustrated. We converted these estimates to annual rates of change for each study area, and multiplied them by the species’ range size within each area. We used the weighted values to calculate an average annual rate of change for each of the 256 permutations, derived from the four change estimates and four study areas. Finally, we calculated the median plus quartiles 1 and 3 from these permutations, and projected these over three generation lengths.

We used the following protocol to estimate each species’ annual rate of change within a given study area. First, we averaged its encounter rates within PAs and UPAs separately during the early (E) and the recent period (R). We weighted each average by the extent of land within PAs and UPAs within the species’ range in the study area in question, extracted from the African Raptor Databank^83^ (Extended Data Fig. 2). Since not all of the selected PAs had been surveyed within a given study area, we estimated each species’ overall encounter rate under two scenarios, in which the average encounter rate within unsurveyed PAs was assumed to have either been (1) the same as in surveyed PAs or (2) the same as in UPAs. These scenarios respectively yielded a high and low estimate of the species’ average encounter rate in each study area and survey period, and hence produced four estimates of change (*C*) between the two periods. These corresponded to E1→R1, E1→R2, E2→R1 and E2→R2. We converted these to annual rates of change using the formula AC = −(1 − (1 + *C*)ˆ(1/*t*)), where ‘AC’ is the annual rate of change, ‘*C*’ is the overall change between the two periods (replaced by each of the four change estimates in turn), and ‘*t*’ is the time (in years) separating the midpoints of the two survey periods. This provided four estimates of the annual rate of change of each species in each study.

Fifteen species had been surveyed adequately in just a single study area. For these, we calculated a median annual rate of change from the four estimates. For each of the remaining 27 species, surveyed in multiple studies, we calculated a median annual rate of change by combining one of the *n* change estimates in turn from each of the relevant studies (Extended Data Fig. 2). Importantly, we weighted each change estimate in accordance with the species’ range size in the study area in question so that extreme changes within a relatively small area (for example, northern Cameroon) did not disproportionately influence the median value. Thus, for species surveyed in two, three or four studies, we calculated a weighted median annual rate of change (AR) from 16, 64 or 256 permutations, respectively. We projected this value (plus quartiles) over three generation lengths (GLs) (ref. ^51^, R. Martin, personal communication, 2021; Supplementary Table 2) using the formula −(1 − (1 + AR)ˆ(3 × GL)).

In the approach described above, we extrapolated mean encounter rates from surveyed PAs and UPAs to unsurveyed PAs, on the assumption that encounter rates within the latter were likely to be similar to those recorded on surveyed land. To test the effects of these extrapolations, we also estimated rates of change when unsurveyed PAs were excluded from the analyses. Change estimates derived from these two approaches typically differed by just 1–2 percentage points over three generation lengths (median = 1.0; range = 0.1–7.4; *n* = 42), supporting our decision to use extrapolated values for unsurveyed PAs (Supplementary Table 5 and Fig. 1). Notably, the exclusion of unsurveyed PAs typically yielded decline rates that were slightly more pronounced than those presented in Table 1 and Fig. 2, suggesting that our decline estimates are slightly conservative.

When combining PA and UPA data from multiple studies, we thus weighted annual change rates by the land area surveyed in each study to produce a composite estimate of each species’ rate of change (Extended Data Fig. 2). It was not possible to apply a similar weighting when comparing PA and UPA rates of change due to differences in the relative area of protected and unprotected land present in each study area. For example, most of the protected and unprotected land surveyed occurred in northern Botswana and West Africa, respectively. Had we applied a weighting based on land area, change rates within PAs would have more strongly reflected conditions in northern Botswana, while those in UPAs would have reflected conditions in West Africa. Since declines were significantly more severe in West Africa, this approach would have exaggerated the apparent benefits of site protection. To avoid this potential bias, we compared PA and UPA change rates using unweighted values.

As a measure of each species’ dependency on protected areas, we compared its encounter rates within PAs and UPAs by subtracting the UPA value from the PA value and dividing by the higher value. Thus, if a species’ mean encounter rate within PAs was higher than in UPAs, we calculated its PA dependency index as: (PA rate − UPA rate)/PA rate. Index values potentially ranged from −1.0 (recorded only in UPAs) to +1.0 (recorded only in PAs).

Median body mass values were extracted from ref. ^84^. In recent African raptor studies, species have been classified as ‘large’ on the basis of a body mass threshold typically set at 1,000–1,400 g^21,23,42,43^. Following ref. ^42^, we adopted 1,300 g as the threshold separating these two size groups, partly reflecting their prey requirements, extracted from ref. ^85^. Among the 42 species surveyed, those weighing ≤1,300 g prey mainly on small mammals, birds, lizards or invertebrates, while the heavier species prey mainly on larger reptiles (particularly snakes), medium-sized birds or mammals, or else scavenge on carcasses (Supplementary Table 2).

We used general linear models (GLMs) and non-parametric tests in R (v.4.1.3)^86^ to examine changes in species encounter rates in relation to survey period, study area, body mass, protected area status and PA dependency. GLMs were run using the ‘lme4’ package. Where the same species or studies were sampled multiple times, the variables ‘Species’ and/or ‘Study’ were included as random terms. Otherwise, measurements were taken from distinct samples. To avoid over-parameterization, we limited the combined number of explanatory and random variables to two (where *n* ≥ 42) or three (*n* ≥ 60). Where appropriate, we compared model variants in which the explanatory variables were either entered separately or as an interaction term. We selected a top model by applying the Akaike information criterion, corrected for small sample sizes (AICc), using ‘AICctab’ in the package ‘bblme’. We used the ‘Anova’ function to calculate Chi-squared and (two-tailed) *P* values for each explanatory term, and applied the functions ‘testUniformity’, ‘testDispersion’, ‘testOutliers’ and ‘testQuantiles’ in the package ‘DHARMa’ to check that the data complied with model assumptions. Where diagnostics indicated a poor model fit, we instead used a paired Wilcoxon signed-rank test or a Kruskal–Wallis test, as appropriate. Analyses are referred to in the results section as models 1 to 17 in Extended Data Table 1, where each model is summarized.

### Determining direction of change from SABAP2 reporting rates

To examine trends among raptors in South Africa, we measured variation in reporting rates during SABAP2 (2007–2021)^46^ using survey data downloaded from ref. ^87^, each entry recording the outcome of one visit to one 5’ × 5’ grid cell (pentad). However, interpreting change in SABAP2 reporting rates (the proportion of pentad survey visits yielding at least one sighting of the target species) is problematic, as rates vary in a nonlinear manner in relation to abundance^88^. We therefore limited our analysis to determining the direction of change. Since relatively few data were collected during 2007, we restricted the dataset to the years 2008–2021. We established that reporting rates tended to increase in relation to visit duration, and decided to limit the dataset to visits of 2–5 h (Supplementary Fig. 2). To ensure adequate survey coverage, we selected pentads that had been surveyed at least 20 times, with a minimum of five visits each in 2008–2014 and 2015–2021. We further limited the dataset to pentads in which the target species was recorded at least twice during the 14-yr period, as confirmation of occupancy. Of the 42 species examined, 30 met these selection criteria within at least 30 pentads in South Africa (Extended Data Table 2).

We used the ‘glmer’ function in R to determine, for each species in turn, whether SABAP2 reporting rates varied significantly in relation to year. We specified the target species’ detection during pentad visits as the dependent variable (binary: positive, negative) and ‘Year’ (numeric: 08–21) as a fixed effect, fitting each model with a binomial error distribution. Since reporting rates tend to vary seasonally, we also entered ‘Seasonal interval’ as a fixed effect, dividing the calendar year into 6, 4, 3, 2 or 1-month intervals in separate model variants. Since each pentad was sampled multiple times, ‘Pentad ID’ was entered as a random effect. We selected a top model on the basis of the minimum AICc value. Where the AICc values for model variants differed by no more than 2 points we selected the variant in which ‘Seasonal interval’ was more finely resolved, for example, into 12 calendar months rather than six 2-month intervals. The direction of change in reporting rates was determined from the slope coefficient, and Chi-squared and *P* values were calculated using the ‘Anova’ function (Extended Data Table 2).

### Reporting summary

Further information on research design is available in the Nature Portfolio Reporting Summary linked to this article.

### Supplementary information

Supplementary Information Extended Methods, Supplementary Tables 1–9, Figs. 1–3 and References.

Reporting Summary

### Source data

Source Data Fig. 1 Source data for regional bar charts, showing percentage decline in bateleur encounter rates within protected and unprotected areas, projected over three generation lengths.

Source Data Fig. 2 Source data for horizontal bar chart, showing percentage change in encounter rates for 42 species, projected over three generation lengths.

Source Data Fig. 3 Source data for bubble chart, showing change rates over three generation lengths, in relation to body mass.

Source Data Fig. 4 Source data for Fig 4 showing: decline rates within regions, in relation to PA status; change rates in relation to body mass and PA status; modelled change rates within PAs and UPAs, in relation to body mass.

Source Data Fig. 5 Source data for Fig. 5 showing change in PA dependency values in relation to body size class, and change in PA dependency in relation to change in abundance.

## Figures and Tables

**Extended Data Table 1:**
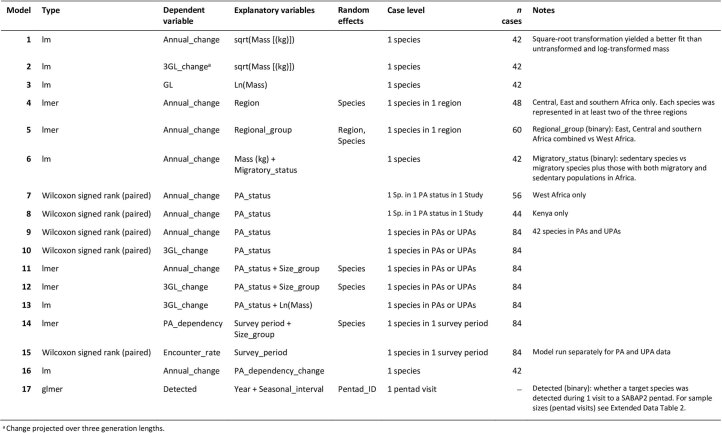
Details of statistical models referred to in the Results section

All models were run in R and produced two-tailed *P* values.

**Extended Data Table 2:**
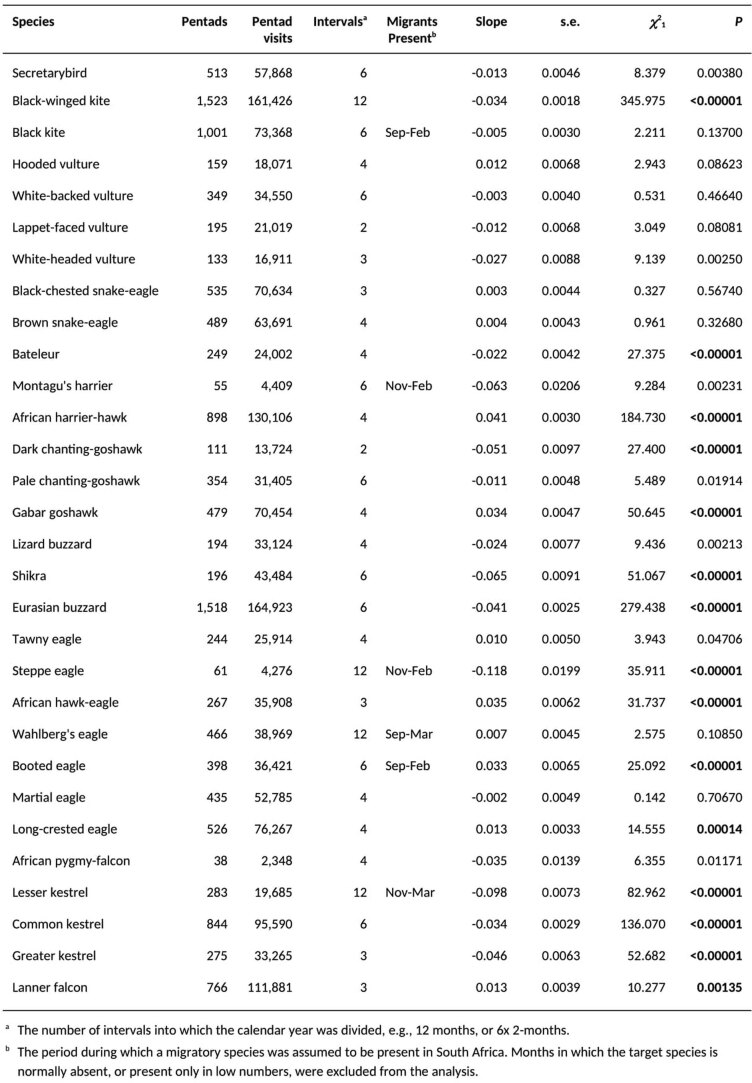
Analysis of SABAP2 reporting rates for 30 raptor species in South Africa during 2008–2021

For data selection criteria and model structure see Methods and model 17 in Extended Data Table 1. The direction of change in reporting rate is given by the slope coefficient. Significant changes are shown in bold (Bonferroni correction applied). Species are ordered taxonomically, as in Table 1.

## Data Availability

Survey data used in statistical analyses are available in Figshare, with the identifier 10.6084/m9.figshare.23727030. Additional background data used in the study are available in the Supplementary Information. Source data are provided with this paper.

## Code availability

Statistical analyses were conducted using open-source packages and functions in R. Copies of the code used to reformat data and perform analyses are available in Figshare, with the identifier 10.6084/m9.figshare.23727030.
