# Supplementary material for: African savanna raptors show evidence of widespread population collapse and a growing dependence on protected areas
Source: Nat Ecol Evol. 2024 Jan 4;8(1):45–56. doi: 10.1038/s41559-023-02236-0 (PMC10781635; doi:10.1038/s41559-023-02236-0)
Supplement: Supplementary file 1 — Extended Methods, Supplementary Tables 1–9, Figs. 1–3 and References. [file 41559_2023_2236_MOESM1_ESM.pdf]

nature ecology & evolution

Article

https://doi.org/10.1038/s41559-023-02236-0

African savanna raptors show evidence of widespread population collapse and a growing dependence on protected areas

In the format provided by the authors and unedited

Supplementary Information: Table of contents

Extended Methods **2**

Road transect analyses **2**

Case selection **3**

Survey routes and protected areas **3**

Comparing protected and unprotected areas **3**

SABAP2 analysis **5**

Anthropogenic pressures **5**

Detectability **6**

**Supplementary Table 1**	The timing and distances covered by road transect surveys from which data were drawn.	**7**
**Supplementary Table 2**	Body mass, diet and generation lengths of study species.	**8**
**Supplementary Table 3**	Encounter rates and total numbers of individuals recorded in each road transect study.	**10**
**Supplementary Table 4**	Designation types included as protected areas in the four road transect studies.	**13**
**Supplementary Table 5**	Comparison of change estimates derived from two methods.	**14**
**Supplementary Table 6**	Biomes and annual precipitation limits of road transect survey areas in each country.	**15**
**Supplementary Table 7**	Species encounter rates on road transects in Botswana, in relation to protected area proximity.	**16**
**Supplementary Table 8**	Changes in human and livestock population densities, and agricultural land area throughout each study period.	**18**
**Supplementary Table 9**	Annual percentage change in woody plant cover in each country surveyed.	**19**
**Supplementary Figure 1**	Effects of excluding unsurveyed PAs when estimating change over three generation lengths.	**20**
**Supplementary Figure 2**	The relationship between SABAP2 reporting rates and visit duration.	**21**
**Supplementary Figure 3**	Raptor encounter rates and rates of change in Botswana, in relation to protected area proximity.	**22**

Supplementary references **23**

Extended Methods

Road transect analyses

Mean encounter rates for each combination of species, survey period and protected area (PA) status were extracted from published sources for West Africa (28 species)[1], Kenya (22 species)[2] and northern Botswana (25 species)[3,4]. Encounter rates for northern Cameroon were calculated from a combination of published[5] and unpublished survey data (15 species), collected in 1973, 2000 and 2007–2010. The latter were made by R.B. and B.M.C., using the same routes and methods as in ref.[5], enabling us to extend the 'recent' survey period in northern Cameroon to span 2000–2010.

In raptor road transect studies, most individual transect surveys yield few or no sightings of a given species. Count data therefore tend to follow a Poisson-like distribution, with high levels of variation between transect surveys, indicating a strong likelihood of over-dispersion, and potentially of zero-inflation. To estimate mean encounter rates during the early (1973) and recent surveys (2000–2010) in northern Cameroon, we used generalised linear mixed effects models (GLMMs), consistent with the approach adopted in Kenya[2] and northern Botswana[4]. Specifically, we used the package *glmmTMB*[6] in R version 3.5.1[7] to model the relationship between survey period, PA status and the number of individuals seen during each transect survey, specifying a Poisson or a negative binomial error distribution and calculating the variance for the latter either as $\varphi\mu$ or as $\mu(1+\mu/k)$ (refs.[6,8]). For each of these three models, we specified a non-zero-inflated and two zero-inflated versions, where the level of zero inflation was either assumed to be constant, or to vary in relation to survey period and PA status, after ref.[6]. We thus compared nine model variants per species.

In each model we entered the number of individuals of the target species per transect survey as the dependent variable. Since changes in abundance between survey periods were likely to differ between protected and unprotected areas, we entered 'Period' and 'PA status' (both binary) as an interaction term. 'Survey year' was fitted as a random term, as some transects had been surveyed more than once in a given year. Since transects varied in length, we entered transect length (log transformed) as an offset term, following refs.[9,10], and used a log-link function throughout.

When examining the effects of period and PA status, we used the package *DHARMa*[11] to identify models showing an acceptable fit, and selected a final model based on minimum AICc value. We used the *Anova* function to calculate Chi-squared and *P*-values for each explanatory term, and applied the R *predict* function to derive the number of encounters predicted for each transect survey. We divided these values by transect length to give a predicted encounter rate (birds 100 km^{-1}), and calculated the mean encounter rate for protected and unprotected areas (UPAs) in each survey period.

Mean encounter rates for raptor species surveyed in northern Botswana during 1991–1995 and 2015–2016 are given in ref.[4], in which separate estimates for PAs and UPAs are provided for only two species, whose change rates differed significantly in relation to PA status. In order to calculate mean encounter rates for all combinations of species, period and PA status in northern Botswana, we applied the same approach as described above for northern Cameroon, and as used for Kenya[2]. In Botswana, the Degree Grid Square (DGS) through which each transect passed had also been recorded, and since some DGSs encompassed multiple transects, we entered 'DGS' as a random term, to account for the lack of independence between such transects.

Case selection

We restricted our analyses to cases in which – within the early survey period – at least five individuals were seen in PAs and five in UPAs, with a minimum of 20 individuals seen in total. These thresholds were met in 90 combinations of species (42) and study area (up to four), for which the median number of individuals encountered in both periods combined was 206 (range: 27–8,193; quartiles: 102–424; Supplementary Table 3). Within the early survey period alone, a median of 32 individuals (quartiles: 17–70) were detected in PAs and 62 (quartiles: 27–136) in UPAs. Sample sizes fell below one or both thresholds in a further 47 cases, including 10 cases in which fewer than five individuals were recorded in PAs and UPAs combined. Cases in which sample sizes failed to meet the above thresholds during the early survey period were excluded from the analysis, to ensure that trend estimates were not disproportionately influenced had 1-2 additional, or fewer, individuals been seen. Furthermore, where initial sample sizes fell below five individuals the apparent direction of change is likely to have been biased – towards recording substantial proportional increases.

Since trend estimation was much less sensitive to the effects of small sample size during the subsequent survey period, minimum sighting thresholds were not applied to recent surveys. This also ensured that cases in which a species had been extirpated, or had become effectively too rare to detect, were retained in the dataset.

In addition, we excluded six cases involving three species: African fish eagle *Haliaeetus vocifer* (all four studies), which is associated mainly with large water bodies and hence poorly captured by road transect studies; and common and lesser kestrel (*Falco tinnunculus* and *F. naumanni*) in West Africa, where these taxa were not always separated at species level[1].

Survey routes and protected areas

Our estimates of the extent of land surveyed in each country were based on biome coverage[12] and annual precipitation levels recorded in areas through which survey routes had passed. Areas in which the biome and average annual precipitation matched those of the routes surveyed were included in the analysis; those in which the dominant biome or precipitation levels differed from those associated with survey routes were excluded (Extended Data Fig. 1, Supplementary Table 6).

The effectiveness of protected area management can differ markedly between PA types and countries, reflecting differences in their aims, national legislation, governance and conservation budgets. PA categories that were considered by study authors to afford little or no meaningful protection for wildlife, or where the degree of protection afforded was uncertain, were excluded from analyses and treated as unprotected. These typically included forest reserves, hunting areas, partial reserves and community conservancies. Across the six countries in which road transects were conducted, the remaining categories (Supplementary Table 4) comprised 168 PAs, of which 44 (26%) were surveyed. Together, surveyed sites accounted for 73% of the land contained within the 168 PAs.

Comparing protected and unprotected areas

Encounter rate differences between protected and unprotected land may potentially have several causes, including inherent differences in habitat suitability that pre-date site designation; a deterioration in habitat suitability outside of PAs, as human pressures intensify; or the positive effects of habitat management and species protection within PAs.

To examine the potentially confounding effects of habitat variation on PA–UPA differences, we re-examined raptor encounter rates from transects surveyed in northern Botswana. Here, the number of transect surveys conducted was large (n = 656) and the boundaries of several major PAs are completely

straight for long distances (Extended Data Fig. 1), bisecting extensive tracts of land that are relatively uniform in terms of vegetation, rainfall and (exceptionally low) human population density (M.H., pers. obs.). Indeed, much of the area falls within the same biome[13], and the habitat mix is essentially similar within PAs and adjacent UPAs (M.H., pers. obs.), minimising any confounding effects of habitat variation.

Since transects surveyed in Botswana had been assigned to their respective 1°x1° (c. 100x100 km) grid squares[3,4], we compared encounter rates in three situations: A. protected areas; B. unprotected areas in squares where PAs were present; C. squares where PAs were absent. We predicted that for most raptor species, encounter rates would be highest on type 'A' and lowest on type 'C' transects. Conversely, we expected rates of decline between survey periods to be lower on type 'A' than type 'C' transects. For both measures we expected intermediate results from type 'B' transects, on the basis that these were likely to be physically closer and ecologically more similar to PA transects than were 'C' transects. Comparisons between 'A' and 'B' transects should therefore provide a more accurate measure of the effects of site protection, while partly controlling for habitat effects.

To make this comparison we modelled encounter rates for 23 species, from which at least five individuals were recorded on each of the three transect types during the early survey period (1990–1995). We estimated encounter rates using the approach described under 'Road transect analyses'. That is, we used generalised linear mixed effects models (*glmmTMB* in R version 3.5.1) in which the number of individuals encountered per transect survey was entered as the dependent variable. Period and PA status (now three categories instead of two) were entered as an interaction term, and grid square as a random term. Transect length (log transformed) was entered as an offset, following refs.[9,10], and a log-link function was used throughout. We used the R *predict* function to derive the number of encounters predicted for each transect survey, from which we calculated encounter rates (birds 100 km^{-1}).

During the early survey period (1990–1995), encounter rates differed significantly between the three transect types (Kruskal-Wallis: $\chi^2_2 = 6.755$, $P = 0.034$, $n = 23$ species). The median encounter rate from type 'A' transects (within PAs) was 1.6 times that of type 'B' transects (in squares with PAs; $P = 0.356$), and 3.2 times that of type 'C' transects (in squares lacking PAs; $P < 0.001$; Bonferroni adjustment applied) (Supplementary Table 7, Fig. 3). In contrast, there was no significant difference in encounter rates overall during the second survey (Kruskal-Wallis $\chi^2_2 = 2.641$, $P = 0.267$), in which the median encounter rate from type 'A' transects was 1.4 times that of type 'B' ($P = 0.634$) and 3.4 times that of type 'C' transects ($P < 0.001$).

Between survey periods, 22% of species showed an increase in encounter rates on type 'A' transects, compared with 35% and 17% on transect types 'B' and 'C' ($\chi^2_2 = 0.363$, $P = 0.834$). The median percentage change was negative in all three categories ('A': -37%; 'B': -33%; 'C': -48%; Supplementary Table 7), but there was no significant effect of transect type overall (Kruskal-Wallis: $\chi^2_2 = 0.639$, $P = 0.726$), or in pairwise comparisons

In conclusion, raptor encounter rates on type 'A' transects were c. 1.4–1.6 times that of type 'B' transects, and 3.2–3.4 times that of type 'C' transects, suggesting that raptor abundance levels within PAs in northern Botswana were approximately 1.5–3.2 times higher than on unprotected land. Similar spatial patterns of variation in raptor numbers in Botswana have been described by Herremans and Herremans-Tonnoeyr[3], based on point counts conducted during 1990–1995. These showed that core areas (>30 km within PA boundaries) supported higher densities than more peripheral protected land. Encounter rates on unprotected land up to 15 km from PA boundaries were just 65–70% of those at the core, dropping to c. 40% at a distance of 15–30 km.

These patterns could reflect variation in habitat suitability in relation to distance from PA boundaries, but are also likely to have been influenced by the dispersal of individual raptors between PAs and adjacent

UPAs. A more targeted study design would be required to separate the direct effects of site protection from those of habitat variation and dispersal, to gauge the effectiveness of PA management on its own. However, we caution that the range and intensity of anthropogenic pressures influencing raptor trends in Botswana are likely to be markedly atypical of Africa as a whole. While the impacts of cattle grazing on vegetation cover – and hence on raptor prey populations – are substantial, the country has an exceptionally low human population density (c. 10% of the continental average; Supplementary Table 8), whose direct effects on raptor populations, through persecution, poisoning, infrastructure development and disturbance, are thought to be small, relative to those in most other African countries[3].

SABAP2 analysis

To determine the direction of change in the abundance of raptor species in South Africa, we examined variation in reporting rates during the second Southern African Bird Atlas Project, spanning 2008–2021[14]. That is, we measured change in the proportion of atlas survey visits during which at least one individual of the target species was recorded. Since longer visits are more likely to yield at least one sighting of a target species, we first investigated the relationship between visit duration and reporting rate for each of the 30 raptor species meeting the selection criteria described in the Methods.

While the mean reporting rate increased sharply between visits of 1 and 2 hours duration, little change was evident for visits of 2–5 hours, after which the mean rate increased gradually (Supplementary Fig. 2). We therefore limited our analysis of SABAP2 data to survey visits lasting 2–5 hours, inclusive.

Anthropogenic pressures

Declines in raptor encounter rates in West Africa (Burkina Faso, Niger and Mali combined) were significantly greater than those recorded in the three remaining regions (Fig. 4a), perhaps reflecting regional variation in human population growth and its attendant pressures. To examine this possibility, we assessed patterns of change in three anthropogenic factors over the lifespan of each study: human population density[15], livestock density[16] and the proportion of land used for agricultural production[17]. Here, we summarise variation in these potential drivers among the countries surveyed, while noting that a full assessment of environmental change in each region lies beyond the scope of this study.

While the human population rose during the course of each study, the annual rate of change recorded in West Africa (+2.7%) lay between the extremes represented by South Africa (+1.3%) and Kenya (+3.2%), and was similar to that of Africa as a whole (+2.6%) (Supplementary Table 8). By 2005, human population densities within the five study areas ranged from 3.2 km^{-2} to 60.2 km^{-2} (Botswana and Kenya, respectively), and were lower in West Africa (14.8 km^{-2}) than the average across Africa (30.5 km^{-2}). This partly reflects the very extensive areas of Sahara-Sindian biome encompassed by Mali and Niger, where the human population is sparse, contrasting with the Sahel and Sudan-Guinea Savanna biomes further to the south, where raptor surveys were conducted. Hence, the average figure for West Africa may have under-estimated densities within the Sahel and Sudan-Guinea Savanna biomes.

Changes in livestock density showed a broadly similar pattern; the annual rate of change recorded in West Africa (+1.9%) lay close to that of Africa as a whole (+1.7%), and between the extremes evident in Botswana (-1.8%) and Cameroon (+2.9%). Similarly, by 2005 the mean livestock density in West Africa (6.9 livestock units km^{-2}) was close to the mean for Africa (6.8), but midway between that of Botswana (2.2) and Kenya (15.9) (Supplementary Table 8). Here again, the relatively low average value recorded for West Africa likely reflects the sparsity of livestock within the Sahara-Sindian biome of northern Mali and Niger. Notwithstanding this effect, West Africa was exceptional in terms of

agricultural expansion during the 1970s–2000s. The annual rate of expansion in agricultural land – comprising arable, permanent crops, other cultivated land and pasture – was more than three times that of Africa as a whole, and almost twice that of any other study area. Although the proportion of agricultural land remained comparatively low in 2005, West Africa's rapid agricultural expansion may partly explain the steep declines evident among its raptor populations. Note, however, that differences in cultural values, belief-based consumption and attitudes to wildlife use will also have contributed to regional differences in raptor population change.

Detectability

Patterns of change in species' encounter rates could have been influenced by changes in their detectability, caused by variation in the height or density of roadside woody vegetation over time. Since vegetation structure was not monitored in the four road transect studies, we were unable to test whether changes in woody cover had occurred on a scale likely to influence detection rates. Instead, we examined evidence from land cover studies, which confirmed that continental-scale changes in forest cover and woody plant encroachment had occurred throughout Africa since the 1970s.

In sub-Saharan Africa, the area of forest and natural non-forest vegetation contracted by 16.3% and 4.7% respectively, during 1975–2000[18], coinciding approximately with the timespan of road transect studies conducted in West Africa, northern Cameroon and Kenya. In contrast, several studies have reported a marked, widespread increase in woody plant encroachment within non-forest natural habitats in parts of Africa[19,20], including an 8% increase within sub-Saharan Africa in 1986–2016[21]. Thus, while the extent of natural non-forest vegetation contracted during 1975–2000[18], woody plant encroachment within this land cover type increased[21].

These changes have been attributed to a range of factors, including overgrazing by livestock, loss of browsing by wild megaherbivores, fire suppression and CO_2 enrichment[19,20,21]. Venter et al.[21] show that the direction and scale of change in woody plant cover has varied markedly between the seven countries included in this study. While woody plant cover declined in Niger and Kenya, it increased in the remaining countries (Supplementary Information Table 9). To gauge whether these changes are likely to have influenced raptor detection rates, we assumed that the annual rates of change in woody plant cover calculated for 1986–2016[21] had occurred throughout the timespan of each raptor study. Had this been the case, woody plant cover in Niger and Kenya would have declined by 3% and 4%, respectively, while rising by 18% in Mali, and by 19% in Botswana (since 1994) (Supplementary Information Table 9). Although woody cover change was highest in Cameroon as a whole (+40%), this figure is unlikely to have been representative of northern Cameroon – where the raptor surveys were conducted – due to its much lower rainfall.

Woody encroachment within savanna habitats may thus have influenced species detectability over the timespan of each survey. However, it is unlikely that a given increase in woody plant cover across the landscape has led to an equivalent reduction in raptor detectability. Thus, the overall 8% increase in woody plant cover recorded within non-forest natural habitats in sub-Saharan Africa during 1986–2016[21] is likely to have been partly offset by the 4.7% contraction in the area of non-forest natural habitats recorded during 1975–2000[18], and was small in comparison with most of the species declines reported here.

Supplementary Table 1 | The timing and distances covered by road transect surveys from which data were drawn. The four studies covered a combined distance of 94,151 km, of which 28% lay within protected areas.

Study area	Early period	Combined distance surveyed		Recent period	Combined distance surveyed		Interval (yrs)[a]	Sources
		PAs	UPAs		PAs	UPAs		
Burkina Faso, Niger, Mali	1969–1973	1030 km	7332 km	2003–2004	1030 km	7332 km	32.5	Ref.[1]
Northern Cameroon	1973	240 km	1119 km	2000–2010	433 km	2985 km	31.0	Ref.[5]; R.B. & B.M.C, unpublished
Northern Botswana	1991–1995	6502 km	22,362 km	2015–2016	4982 km	15,730 km	21.9	Refs.[3,4]
Kenya	1970–1977	6464 km	2195 km	2003–2020	5497 km	8918 km	40.0	Refs.[2,22,23]
Totals		14,236 km	33,008 km		11,942 km	34,965 km		

[a] Time interval separating the mid-points of the 'early' and 'recent' survey periods.

Supplementary Table 2| Body mass, diet and generation lengths of study species. Species are listed in descending order of mass. Size classes were based on mass and diet, with the majority of those species assigned to the 'large' category (≥ 1300 g) being dependent on medium-sized mammals, birds or reptiles, or on carrion.

**Species**		**Near/ endemic**[a]	**Group**[b]	**Diet**[c]	**Median mass (g)**[d]	**Generation length (yrs)**[e]
Large raptors						
Rüppell's vulture	*Gyps rueppelli*	Y	Vultures	C	7900	14.4
Lappet-faced vulture	*Torgos tracheliotos*	Y	Vultures	C	6780	14.7
White-backed vulture	*Gyps africanus*	Y	Vultures	C	5675	13.2
Martial eagle	*Polemaetus bellicosus*	Y	Eagles	B/M/R	4605	11.8
White-headed vulture	*Trigonoceps occipitalis*	Y	Vultures	C	4300	10.4
Secretarybird	*Sagittarius serpentarius*	Y	Secretarybird	R/I/M/B	3285	10.1
Steppe eagle	*Aquila nipalensis*		Eagles	I/SM/SB	3175	14.0
Bateleur	*Terathopius ecaudatus*	Y	Snake-eagles	M/B/R/C	2385	14.7
Tawny eagle	*Aquila rapax*		Eagles	M/B/L/C	2350	13.7
Hooded vulture	*Necrosyrtes monachus*	Y	Vultures	C/S	2050	13.0
Brown snake-eagle	*Circaetus cinereus*	Y	Snake-eagles	R/SM/B	2000	9.1
Beaudouin's snake-eagle	*Circaetus beaudouini*	Y	Snake-eagles	R/SM/I	1750	8.7
Black-chested snake-eagle	*Circaetus pectoralis*	Y	Snake-eagles	R/SM/I	1719	8.7
Short-toed snake-eagle	*Circaetus gallicus*		Snake-eagles	R/M	1700	8.9
African hawk-eagle	*Aquila spilogaster*	Y	Eagles	M/B	1425	10.5
Small–medium raptors						
Long-crested eagle	*Lophaetus occipitalis*	Y	Eagles	SM/B/L	1291	7.8
Augur buzzard	*Buteo augur*	Y	Buzzards	R/SM/SB	1110	8.6
Eurasian buzzard	*Buteo buteo*		Buzzards	SM/SB/I	863	9.4
Black kite	*Milvus migrans*		Kites	SM/SB/L/I/C	847	9.3
Booted eagle	*Hieraaetus pennatus*		Eagles	B/SM/I	842	7.0
Wahlberg's eagle	*Hieraaetus wahlbergi*	Y	Eagles	L/B/SM	838	7.6
Pale chanting-goshawk	*Melierax canorus*	Y	Chanting-goshawks	SM/SB/L/I	811	7.8
Dark chanting-goshawk	*Melierax metabates*	Y	Chanting-goshawks	SM/SB/L/I	758	7.5
Western marsh-harrier	*Circus aeruginosus*		Harriers	SM/SB	659	6.5
Lanner falcon	*Falco biarmicus*		Falcons	SB/SM	658	5.0
African harrier-hawk	*Polyboroides typus*	Y	Harrier-hawk	SB/SM/L	652	7.3
Eastern chanting-goshawk	*Melierax poliopterus*	Y	Chanting-goshawks	L/B/SM/I	642	7.5

Species		Near/ endemic[a]	Group[b]	Diet[c]	Median mass (g)[d]	Generation length (yrs)[e]
Grasshopper buzzard	*Butastur rufipennis*	Y	Buzzards	I/SB/SM	340	5.3
Montagu's harrier	*Circus pygargus*		Harriers	SM/I	308	5.6
Lizard buzzard	*Kaupifalco monogrammicus*	Y	Chanting-goshawks	L/I/SB	288	5.8
Fox kestrel	*Falco alopex*	Y	Falcons	SM/SB	275	4.3
Black-winged kite	*Elanus caeruleus*		Kites	SM/I	259	4.7
Grey kestrel	*Falco ardosiaceus*	Y	Falcons	SB/SM	239	4.1
Greater kestrel	*Falco rupicoloides*	Y	Falcons	SM/I	236	4.0
Common kestrel	*Falco tinnunculus*		Falcons	SM/I	214	5.3
Dickinson's kestrel	*Falco dickinsoni*	Y	Falcons	SM/SB/I	204	3.9
Red-necked falcon	*Falco ruficollis*		Falcons	SB/I/SM/L	203	4.4
Shikra	*Accipiter badius*		Sparrowhawks	SB/L	172	4.3
Gabar goshawk	*Micronisus gabar*	Y	Chanting-goshawks	SB/SM/I	168	4.9
Lesser kestrel	*Falco naumanni*		Falcons	I/SM	152	3.8
Scissor-tailed kite	*Chelictinia riocourii*	Y	Kites	I/SM	110	3.8
African pygmy-falcon	*Polihierax semitorquatus*	Y	Falcons	I/SB	58	4.4

[a] Endemic or near-endemic to the African continent.

[b] Groups to which species were assigned in Fig. 3.

[c] Diet, from refs.[24,25]: B, medium-sized birds, e.g., gamebirds, doves; C, carrion; L, lizards; M, medium-sized mammals; I, invertebrates; R, reptiles, including snakes; S, scavenger; SB, small birds; SM, small mammals.

[d] Median of body mass values given in ref.[25].

[e] Estimated generation length (ref.[26]; R. Martin in litt. 2021).

Supplementary Table 3| Encounter rates and total numbers of individuals recorded in each road transect study. Encounter rates are shown in relation to protected area status and survey period, and were derived from 53,209 sightings of the 42 study species.

				Encounter rate (birds 100 km^{-1})			
				Protected		Unprotected	
**Species**	**Study area**	**Surveys**[a]	**Sightings**	**Early**	**Recent**	**Early**	**Recent**
Secretarybird	Kenya	1972, 2012	256	1.32	1.12	5.91	0.24
Sagittarius serpentarius	N. Botswana	1994, 2016	72	0.52	0.20	0.10	0.02
Black-winged kite	West Africa	1971, 2004	189	1.30	0.20	2.00	1.30
Elanus caeruleus	N. Cameroon	1973, 2004	255	4.17	11.90	3.30	11.23
	Kenya	1972, 2012	322	0.51	2.97	4.43	1.03
	N. Botswana	1994, 2016	127	1.42	0.22	0.23	0.09
Scissor-tailed kite	West Africa	1971, 2004	663	2.30	0.10	10.60	1.70
Chelictinia riocourii	N. Cameroon	1973, 2004	461	9.17	192.81	9.61	6.91
Black/ yellow-billed kite	West Africa	1971, 2004	4,698	8.60	4.70	58.30	17.50
Milvus migrans	N. Cameroon	1973, 2004	2,801	167.50	388.39	84.14	35.48
	Kenya	1972, 2012	859	0.54	0.68	17.54	8.43
	N. Botswana	1994, 2016	4,785	25.87	5.55	7.08	4.33
Hooded vulture	West Africa	1971, 2004	8,193	23.60	16.20	84.40	46.40
Necrosyrtes monachus	N. Cameroon	1973, 2004	1,532	24.47	5.74	55.03	31.24
	Kenya	1972, 2012	141	2.80	1.41	2.44	0.19
	N. Botswana	1994, 2016	189	0.43	0.00	0.66	0.29
White-backed vulture	West Africa	1971, 2004	893	30.80	15.40	9.80	0.30
Gyps africanus	N. Cameroon	1973, 2004	438	59.54	34.48	12.16	3.08
	Kenya	1972, 2012	1,895	37.93	18.21	21.20	4.52
	N. Botswana	1994, 2016	4,347	8.67	11.61	10.35	8.37
Rüppell's vulture	West Africa	1971, 2004	2,646	3.40	2.80	61.30	2.50
Gyps rueppelli	N. Cameroon	1973, 2004	383	56.95	10.67	10.80	2.40
	Kenya	1972, 2012	631	11.04	7.14	2.48	2.18
Lappet-faced vulture	West Africa	1971, 2004	130	1.80	1.10	2.90	0.10
Torgos tracheliotos	Kenya	1972, 2012	222	3.76	2.15	1.38	0.36
	N. Botswana	1994, 2016	270	0.84	0.59	0.64	0.29
White-headed vulture	West Africa	1971, 2004	80	4.40	1.30	1.20	0.00
Trigonoceps occipitalis	N. Botswana	1994, 2016	73	0.50	0.04	0.17	0.09
Short-toed snake-eagle *Circaetus gallicus*	N. Cameroon	1973, 2004	102	13.08	7.68	1.68	1.26
Beaudouin's snake-eagle *Circaetus beaudouini*	West Africa	1971, 2004	95	4.40	1.90	1.40	0.10
Black-chested snake-eagle	Kenya	1972, 2012	131	0.83	0.62	0.59	0.33
Circaetus pectoralis	N. Botswana	1994, 2016	123	0.35	0.38	0.16	0.30
Brown snake-eagle	West Africa	1971, 2004	108	2.20	1.00	1.40	0.20
Circaetus cinereus	N. Cameroon	1973, 2004	30	2.08	0.00	1.43	0.37
	Kenya	1972, 2012	278	2.58	1.66	0.23	0.21
	N. Botswana	1994, 2016	258	0.90	0.70	0.35	0.65

				Encounter rate (birds 100 km^{-1})			
				Protected		Unprotected	
Species	Study area	Surveys[a]	Sightings	Early	Recent	Early	Recent
Bateleur	West Africa	1971, 2004	395	18.50	11.60	2.40	0.00
Terathopius ecaudatus	N. Cameroon	1973, 2004	121	28.72	11.44	1.81	0.19
	Kenya	1972, 2012	1,427	12.88	8.18	1.13	0.48
	N. Botswana	1994, 2016	1,300	6.17	4.04	2.84	1.25
Western marsh-harrier *Circus aeruginosus*	West Africa	1971, 2004	393	3.90	4.90	3.40	3.10
Montagu's harrier	West Africa	1971, 2004	294	2.80	1.20	3.60	0.90
Circus pygargus	N. Cameroon	1973, 2004	237	5.00	24.65	4.95	5.39
	Kenya	1972, 2012	410	1.09	7.51	7.69	0.17
African harrier-hawk	West Africa	1971, 2004	37	2.10	1.10	0.60	0.10
Polyboroides typus	N. Botswana	1994, 2016	36	0.15	0.05	0.07	0.06
Dark chanting-goshawk	West Africa	1971, 2004	393	3.50	2.70	3.60	1.50
Melierax metabates	N. Cameroon	1973, 2004	240	15.42	13.20	9.54	4.43
	N. Botswana	1994, 2016	82	0.16	0.10	0.17	0.14
Eastern chanting-goshawk *Melierax poliopterus*	Kenya	1972, 2012	822	3.96	5.87	0.71	3.66
Pale chanting-goshawk *Melierax canorus*	N. Botswana	1994, 2016	428	1.49	2.19	0.50	0.66
Gabar goshawk	West Africa	1971, 2004	236	2.80	3.00	2.10	1.10
Micronisus gabar	N. Botswana	1994, 2016	127	0.41	0.69	0.28	0.15
Lizard buzzard *Kaupifalco monogrammicus*	West Africa	1971, 2004	61	4.90	2.90	0.60	0.40
Shikra	West Africa	1971, 2004	153	6.80	6.00	1.30	0.30
Accipiter badius	N. Botswana	1994, 2016	111	0.59	0.10	0.24	0.04
Grasshopper buzzard *Butastur rufipennis*	West Africa	1971, 2004	303	9.50	6.40	3.50	1.50
Eurasian buzzard	Kenya	1972, 2012	102	0.25	1.87	0.57	0.68
Buteo buteo	N. Botswana	1994, 2016	235	0.57	0.43	0.71	0.37
Augur buzzard *Buteo augur*	Kenya	1972, 2012	674	0.25	1.23	21.77	1.88
Tawny eagle	West Africa	1971, 2004	178	2.30	0.80	1.60	0.20
Aquila rapax	N. Cameroon	1973, 2004	88	13.81	9.76	1.68	0.49
	Kenya	1972, 2012	674	2.49	5.79	2.51	2.05
	N. Botswana	1994, 2016	467	1.35	1.63	0.70	1.04
Steppe eagle	N. Cameroon	1973, 2004	56	7.35	4.45	1.10	0.57
Aquila nipalensis	Kenya	1972, 2012	189	6.53	2.67	1.16	0.15
	N. Botswana	1994, 2016	62	0.31	0.03	0.23	0.05
African hawk-eagle	West Africa	1971, 2004	45	3.00	0.90	0.80	0.10
Aquila spilogaster	N. Botswana	1994, 2016	45	0.33	0.09	0.12	0.00
Wahlberg's eagle	West Africa	1971, 2004	53	3.10	2.30	1.10	0.00
Hieraaetus wahlbergi	Kenya	1972, 2012	344	2.97	1.95	1.11	0.52
	N. Botswana	1994, 2016	88	0.29	0.08	0.20	0.11
Booted eagle *Hieraaetus pennatus*	West Africa	1971, 2004	186	2.00	1.60	1.40	1.50
Martial eagle	West Africa	1971, 2004	27	1.40	0.70	0.80	0.00
Polemaetus bellicosus	Kenya	1972, 2012	111	0.53	1.29	0.22	0.08
	N. Botswana	1994, 2016	68	0.16	0.10	0.18	0.11

Species	Study area	Surveys[a]	Sightings	Encounter rate (birds 100 km^{-1})			
				Protected		Unprotected	
				Early	Recent	Early	Recent
Long-crested eagle	N. Cameroon	1973, 2004	37	4.58	1.17	1.67	0.39
Lophaetus occipitalis	Kenya	1972, 2012	153	0.15	0.86	6.17	0.29
African pygmy-falcon *Polihierax semitorquatus*	Kenya	1972, 2012	170	0.92	1.72	0.18	0.63
Lesser kestrel	Kenya	1972, 2012	829	2.42	10.30	23.89	0.84
Falco naumanni	N. Botswana	1994, 2016	109	1.42	0.09	0.07	0.01
Common kestrel	N. Cameroon	1973, 2004	375	20.00	10.19	23.72	2.79
Falco tinnunculus	Kenya	1972, 2012	187	1.28	1.04	3.85	0.07
Greater kestrel *Falco rupicoloides*	N. Botswana	1994, 2016	531	1.23	1.08	1.03	0.83
Fox kestrel *Falco alopex*	West Africa	1971, 2004	279	0.90	1.20	5.90	2.10
Grey kestrel *Falco ardosiaceus*	West Africa	1971, 2004	143	5.60	4.40	1.00	0.40
Dickinson's kestrel *Falco dickinsoni*	N. Botswana	1994, 2016	28	0.27	0.15	0.09	0.01
Red-necked falcon *Falco ruficollis*	West Africa	1971, 2004	75	1.10	0.20	1.50	0.70
Lanner falcon	West Africa	1971, 2004	225	1.30	0.80	1.90	1.20
Falco biarmicus	N. Botswana	1994, 2016	94	0.48	0.35	0.18	0.12

[a] The midpoint of each survey period.

Supplementary Table 4| Designation types included as protected areas in the four road transect studies. Designation names were taken from the World Database on Protected Areas[27].

Designation	Mali	Burkina Faso	Niger	Northern Cameroon	Kenya	Northern Botswana
National Park	+	+	+	+	+	+
Game Reserve			+			+
Bird Sanctuary	+					
Classified Forest (including Partial & Total Wildlife Reserves)		+				
Forest Reserve						+
Hunting Area	+					
National Reserve					+	
Nature Reserve			+			
Partial Fauna Reserve			+			
Partial Wildlife Reserve		+				
Private Protected Areas, Ranches and Reserves					+	
Strict Nature Reserve			+			
Total Wildlife Reserve	+					

Supplementary Table 5| Comparison of change estimates derived from two methods. We estimated each species' rate of change from two alternative scenarios, in which its encounter rates within unsurveyed PAs were assumed to have been the same as in surveyed PAs, or the same as in UPAs. We calculated median, Q1 and Q3 change rates, over three generations, based on these extrapolations (Table 1). Below, we compare the results from this approach ('Extrapolation') with those from an alternative, in which unsurveyed PAs were excluded from the analysis ('No extrapolation'). Change estimates from the two approaches typically differed by just 1–2 percentage points (median: 1.0; range: 0.1–7.4; *n* = 42). Disparities between the two approaches are illustrated in Supplementary Fig. 1.

	**No extrapolation**	**Extrapolation**				
**Species**		**Median**	**Minimum**	**Q1**	**Q3**	**Maximum**
Secretarybird	**-85.7%**	**-85.4%**	-86.0%	-85.7%	-85.1%	-84.7%
Black-winged kite	**-30.8%**	**-32.2%**	-33.2%	-32.6%	-31.7%	-31.1%
Scissor-tailed kite	**-46.9%**	**-47.6%**	-49.2%	-48.1%	-47.1%	-46.0%
Black kite	**-60.3%**	**-60.3%**	-62.2%	-61.0%	-59.6%	-58.3%
Hooded vulture	**-68.3%**	**-67.1%**	-69.6%	-68.0%	-66.2%	-64.5%
White-backed vulture	**-89.0%**	**-86.2%**	-90.2%	-89.6%	-81.8%	-80.7%
Rüppell's vulture	**-97.5%**	**-97.4%**	-97.6%	-97.6%	-97.3%	-97.2%
Lappet-faced vulture	**-91.4%**	**-90.2%**	-92.4%	-92.0%	-88.0%	-87.4%
White-headed vulture	**-92.3%**	**-90.0%**	-93.4%	-93.0%	-85.6%	-84.7%
Short-toed snake-eagle	**-24.8%**	**-25.0%**	-26.7%	-25.6%	-24.4%	-23.3%
Beaudouin's snake-eagle	**-84.9%**	**-83.0%**	-85.9%	-85.3%	-80.4%	-79.5%
Black-chested snake-eagle	**15.9%**	**15.0%**	13.7%	14.3%	15.6%	16.3%
Brown snake-eagle	**-55.4%**	**-54.6%**	-58.7%	-56.9%	-52.3%	-50.1%
Bateleur	**-91.7%**	**-87.1%**	-94.2%	-92.8%	-76.9%	-71.4%
Western marsh-harrier	**-5.1%**	**-4.0%**	-5.9%	-5.3%	-2.6%	-2.0%
Montagu's harrier	**-52.4%**	**-51.1%**	-52.6%	-51.9%	-50.2%	-49.5%
African harrier-hawk	**-60.2%**	**-57.6%**	-63.6%	-61.5%	-53.3%	-50.6%
Dark chanting-goshawk	**-42.1%**	**-41.2%**	-42.4%	-42.3%	-40.0%	-39.9%
Eastern chanting-goshawk	**123.2%**	**115.8%**	104.3%	106.8%	125.3%	128.0%
Pale chanting-goshawk	**39.7%**	**39.7%**	38.2%	39.3%	40.2%	41.3%
Gabar goshawk	**-22.1%**	**-20.9%**	-23.1%	-22.5%	-19.3%	-18.6%
Lizard buzzard	**-20.3%**	**-20.7%**	-27.0%	-22.6%	-18.7%	-13.8%
Shikra	**-48.6%**	**-44.8%**	-51.9%	-49.3%	-39.8%	-36.6%
Grasshopper buzzard	**-33.3%**	**-31.6%**	-37.6%	-34.4%	-28.7%	-25.1%
Eurasian buzzard	**-32.5%**	**-30.9%**	-31.9%	-31.4%	-30.3%	-29.8%
Augur buzzard	**-78.6%**	**-78.4%**	-78.9%	-78.7%	-78.0%	-77.8%
Tawny eagle	**-66.4%**	**-66.3%**	-70.6%	-69.6%	-62.7%	-61.4%
Steppe eagle	**-91.1%**	**-90.8%**	-91.7%	-91.3%	-90.2%	-89.7%
African hawk-eagle	**-91.8%**	**-91.5%**	-92.1%	-91.8%	-91.1%	-90.8%
Wahlberg's eagle	**-80.7%**	**-73.8%**	-82.7%	-81.9%	-62.2%	-60.5%
Booted eagle	**4.3%**	**3.3%**	1.8%	2.1%	4.4%	4.7%
Martial eagle	**-93.1%**	**-89.8%**	-93.9%	-93.6%	-84.0%	-83.1%
Long-crested eagle	**-79.4%**	**-78.7%**	-79.6%	-79.1%	-78.4%	-77.8%
African pygmy-falcon	**45.6%**	**44.0%**	38.9%	41.3%	46.7%	49.3%
Lesser kestrel	**-66.2%**	**-65.3%**	-66.4%	-66.0%	-64.7%	-64.3%
Common kestrel	**-72.0%**	**-70.4%**	-72.3%	-72.0%	-68.8%	-68.5%
Greater kestrel	**-10.6%**	**-10.6%**	-10.7%	-10.6%	-10.5%	-10.5%
Fox kestrel	**-33.6%**	**-33.0%**	-34.8%	-33.9%	-32.1%	-31.2%
Grey kestrel	**-27.3%**	**-24.6%**	-33.7%	-29.0%	-19.8%	-14.1%
Dickinson's kestrel	**-53.3%**	**-53.2%**	-53.4%	-53.3%	-53.1%	-53.0%
Red-necked falcon	**-26.5%**	**-26.9%**	-27.8%	-27.5%	-26.4%	-26.0%
Lanner falcon	**-19.9%**	**-19.9%**	-20.9%	-20.2%	-19.6%	-18.9%

Supplementary Table 6| Biomes and annual precipitation limits of road transect survey areas in each country. Biome boundaries were extracted from ref.[12].

Country	Biomes	Internal boundaries
Mali	Sahel, Sudan-Guinea Savanna	Northern limit defined by the boundary between the Sahel and Sahara-Sindian biomes.
Burkina Faso	Sahel, Sudan-Guinea Savanna	None: whole country lies within these two biomes
Niger	Sahel, Sudan-Guinea Savanna	Northern limit defined by the boundary between the Sahel and Sahara-Sindian biomes.
N. Cameroon	Sudan zone	Southern limit defined by the boundary between the Sudan and Guinea Savanna zones.
Kenya	Somali Masai, Lake Victoria Basin, Sudan-Guinea Savanna, Afrotropical Highlands	Mean annual rainfall: >=300mm, <=1700mm
N. Botswana	Zambezian, Kalahari-Highveld	Mean annual rainfall: >=300mm

Supplementary Table 7| Species encounter rates on road transects in Botswana, in relation to protected area proximity. Encounter rates (birds 100 km^{-1}) during surveys in 1990–1995 and 2015–2016 were calculated for 23 species, from road transects categorised as: A. protected areas; B. unprotected areas in 100x100 km grid squares where PAs were present; C. squares where PAs were absent. Encounter rates on type 'A' transects (protected) were significantly higher than on type 'C' transects (unprotected), in both survey periods. Differences in encounter rates and rates of change on 'A' and 'B' transects were not significant.

Species	Early survey period			Recent survey period			Rates of change		
	Protected (A)	Unprotected (B)	Unprotected (C)	Protected (A)	Unprotected (B)	Unprotected (C)	Protected (A)	Unprotected (B)	Unprotected (C)
Secretarybird	0.52	0.16	0.07	0.19	0.03	0.01	-62%	-84%	-82%
Black-winged kite	1.42	0.36	0.18	0.21	0.06	0.10	-85%	-84%	-42%
Black kite	25.51	3.10	10.15	5.07	4.99	2.92	-80%	61%	-71%
Hooded vulture	0.42	1.60	0.09	0.00	0.75	0.05	-100%	-53%	-39%
White-backed vulture	8.61	14.71	7.17	11.20	17.23	4.54	30%	17%	-37%
Lappet-faced vulture	0.84	1.06	0.45	0.59	0.50	0.17	-30%	-53%	-63%
Black-chested snake-eagle	0.34	0.16	0.16	0.35	0.32	0.30	4%	106%	87%
Brown snake-eagle	0.90	0.69	0.15	0.69	1.01	0.46	-23%	46%	205%
Bateleur	6.21	4.53	1.68	3.93	3.03	0.55	-37%	-33%	-68%
African harrier-hawk	0.14	0.11	0.04	0.05	0.14	0.02	-63%	18%	-61%
Dark chanting-goshawk	0.16	0.12	0.18	0.11	0.17	0.14	-29%	46%	-22%
Pale chanting-goshawk	1.49	0.47	0.54	2.18	0.25	0.87	46%	-46%	60%
Gabar goshawk	0.33	0.33	0.19	0.65	0.27	0.08	98%	-18%	-55%
Shikra	0.65	0.29	0.33	0.11	0.06	0.03	-83%	-79%	-91%
Eurasian buzzard	0.57	0.99	0.58	0.43	0.42	0.35	-25%	-57%	-40%

Species		Early survey period			Recent survey period			Rates of change	
	Protected (A)	Unprotected (B)	Unprotected (C)	Protected (A)	Unprotected (B)	Unprotected (C)	Protected (A)	Unprotected (B)	Unprotected (C)
Tawny eagle	1.41	0.85	0.58	1.59	1.45	0.85	12%	72%	46%
Steppe eagle	0.21	0.41	0.17	0.04	0.07	0.05	-78%	-84%	-68%
African hawk-eagle	0.23	0.31	0.04	0.10	0.00	0.00	-56%	-100%	-100%
Wahlberg's eagle	0.29	0.25	0.17	0.08	0.17	0.09	-72%	-33%	-48%
Martial eagle	0.16	0.35	0.09	0.10	0.22	0.06	-39%	-37%	-40%
Lesser kestrel	1.42	0.08	0.07	0.08	0.00	0.02	-94%	-100%	-76%
Greater kestrel	1.23	0.97	1.07	1.08	0.78	0.87	-12%	-19%	-19%
Lanner falcon	0.47	0.16	0.19	0.35	0.19	0.09	-26%	18%	-53%
**Median:**	**0.57**	**0.36**	**0.18**	**0.35**	**0.25**	**0.10**	**-37%**	**-33%**	**-48%**
**Quartiles:**	0.31, 1.41	0.21, 0.98	0.12, 0.56	0.10, 0.89	0.10, 0.77	0.05, 0.50	–18%, –75%	18%, –68%	–29%, –68%

Supplementary Table 8| Changes in human and livestock population densities, and agricultural land area throughout each study period. Annual rates of change in density are shown for each country or region surveyed for raptors. Values for the African continent (1970–2005) are provided for context. Since survey periods differed between raptor studies, densities are given for a single year (2005), which falls within or near to the end of each study period, to illustrate the degree of variation between study areas.

Country/ region	Time span[a]	Interval (years)	Humans[b]		Livestock[c]		Agricultural land[d]	
			Annual change[e]	Density in 2005 (km^{-2})	Annual change[e]	Density in 2005 (km^{-2})	Annual change[e]	Percentage cover in 2005[f]
Botswana	1994–2016	22	2.1%	3.2	-1.8%	2.2	-0.02%	46%
Cameroon	1973–2004	31	2.9%	36.3	2.9%	8.1	0.38%	19%
Kenya	1972–2012	40	3.2%	60.2	2.5%	15.9	0.23%	47%
West Africa	1971–2004	33	2.7%	14.8	1.9%	6.9	0.75%	30%
South Africa	2008–2021	12	1.3%	39.8	-0.9%	10.4	-0.06%	80%
Africa	1970–2005	35	2.6%	30.5	1.7%	6.8	0.23%	37%

[a] The mid-point of each survey period (N. Botswana, N. Cameroon, Kenya, West Africa). In South Africa, although SABAP2 data spanned 2008–2021, livestock density data were available only up to 2020.

[b] Change in human population estimates, extracted from ref.[15].

[c] The combined density of livestock units for cattle, goats, sheep and camels ref.[16].

[d] Land that is either arable, under permanent crops, meadows or pastures, or cultivated and natural growing ref.[17].

[e] Annual rate of change across the time span of each study. The start and end values were taken as the average of three consecutive years, e.g. 1994, for 1993–1995.

[f] The area of agricultural land expressed as a percentage of the land area of the country or region.

Supplementary Table 9| Annual percentage change in woody plant cover in each country surveyed. The annual rate of woody plant encroachment recorded in each survey country has been estimated for 1986–2016[21]. Here, we have applied these annual rates to the full timespan over which surveys were conducted, e.g. dating back to 1971 in the case of West Africa, to estimate overall change in woody plant cover between 'early' and 'recent' surveys.

Country	Percentage change p.a.	Mid-points of survey periods	Survey timespan (years)	Estimated change in cover over survey timespan
Mali	0.5%	1971, 2004	32.5	18%
Burkina Faso	0.4%	1971, 2004	32.5	14%
Niger	-0.1%	1971, 2004	32.5	-3%
Cameroon[a]	1.1%	1973, 2004	31.0	40%
Kenya	-0.1%	1972, 2012	40.0	-4%
Botswana	0.8%	1994, 2016	21.9	19%
South Africa	0.4%	2008, 2021	14.0	6%

[a] Change in woody plant cover for Cameroon as a whole is likely to have exceeded that in the more arid Sudan Zone of Northern Cameroon.

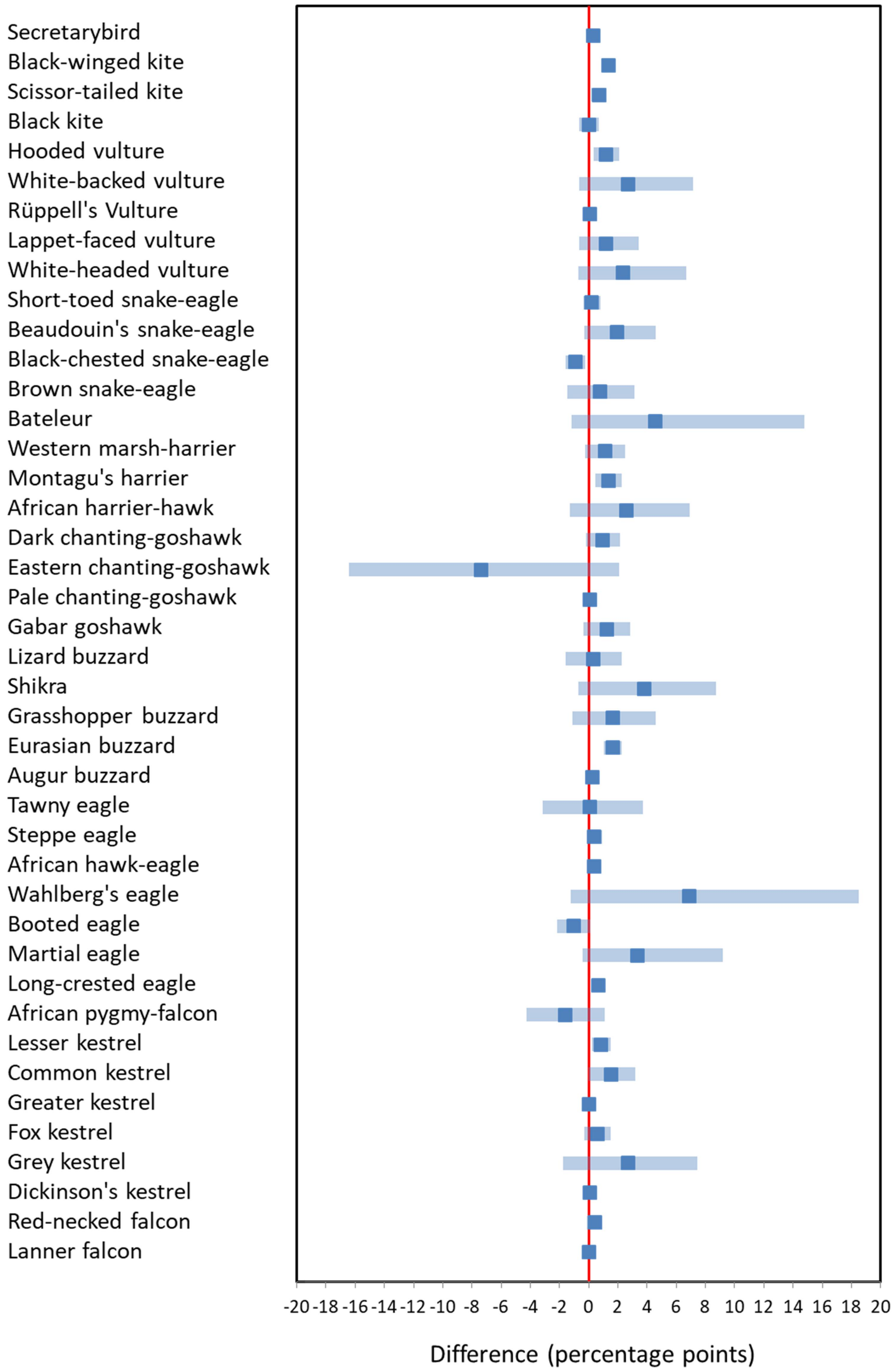

Supplementary Figure 1| Effects of excluding unsurveyed PAs when estimating change over three generation lengths. We compared change estimates obtained from two methods: 'Extrapolation', in which encounter rates within unsurveyed PAs were assumed to have been the same as in surveyed PAs, or the same as in UPAs; and 'No extrapolation', in which unsurveyed PAs were excluded from the analysis (Supplementary Table 5). Estimates from the 'No extrapolation' method are indicated by the vertical red line, around which median, Q1 and Q3 values from the 'Extrapolation' method are shown (dark blue box and horizontal bars, respectively). In most cases, disparities were small (median = 1.0 percentage point; *n* = 42 species) and positive, indicating that decline estimates derived from the 'Extrapolation' method (used in Table 1 & Fig. 2) were often more conservative than those from the 'No extrapolation' method.

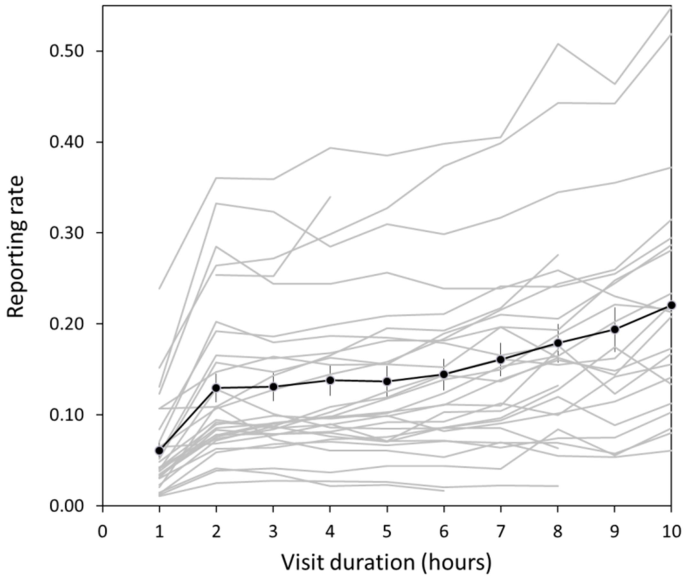

Supplementary Figure 2| The relationship between SABAP2 reporting rates and visit duration. Grey lines indicate species (*n* = 30), and black points show the mean (±1 SEM) reporting rate across all species.

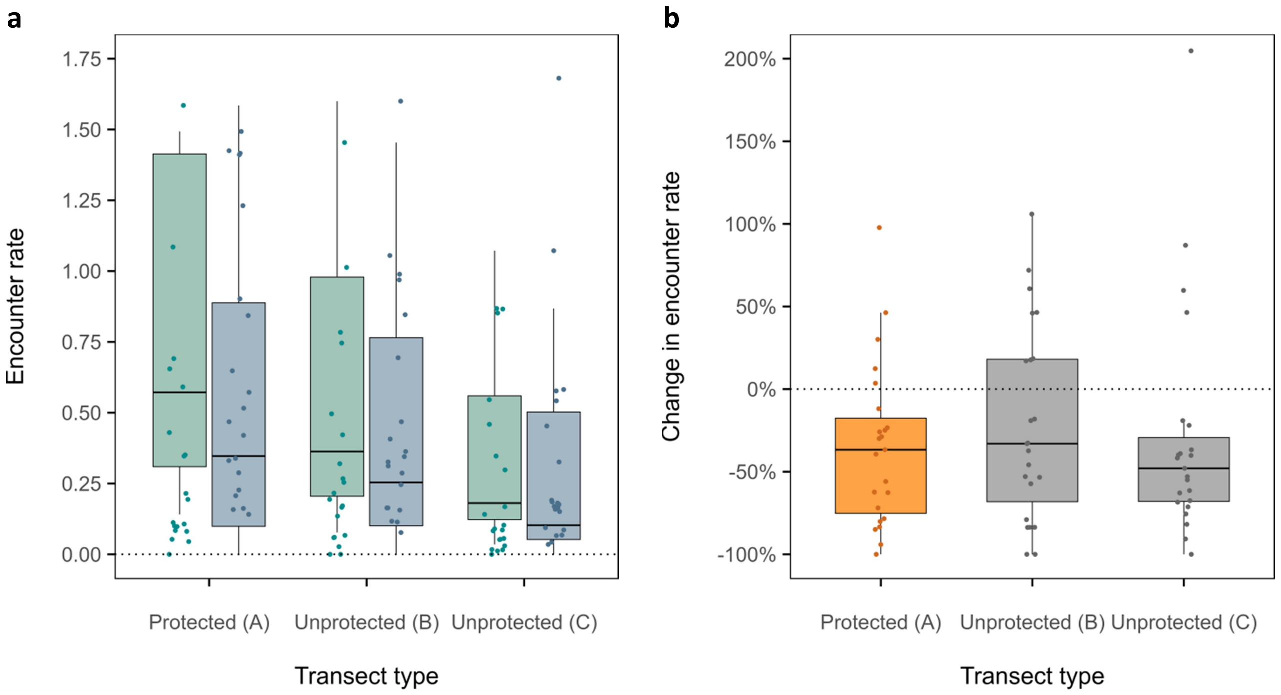

Supplementary Figure 3| Raptor encounter rates and rates of change in Botswana, in relation to protected area proximity. Road transects were categorised as: A. protected areas; B. unprotected areas in 100x100 km grid squares where PAs were present; C. squares where PAs were absent. **a**, During surveys in 1990–1995 (green) and 2015–2016 (blue), encounter rates differed in relation to transect type, being significantly higher on type 'A' than type 'C' transects (*n* = 23 species assessed). There were no significant differences between encounter rates on type 'A' and 'B' transects. **b**, Change in encounter rates within protected (orange) and unprotected areas (grey), was unrelated to transect type. Boxplots show the median, first and third quartiles. Whiskers extend to ±1.5 x the inter-quartile range; points represent individual species.

References

1. Thiollay, J.M. The decline of raptors in West Africa: long-term assessment and the role of protected areas. *Ibis* **148**, 240–254 (2006).
2. Ogada, D. et al. Evidence of widespread declines in Kenya's raptor populations over a 40-year period. *Biol. Conserv.* **266**, 109361 (2022).
3. Herremans, M. & Herremans-Tonnoeyr, D. Land use and the conservation status of raptors in Botswana. *Biol. Conserv*. **94**, 31–41 (2000).
4. Garbett, R., Herremans, M., Maude, G., Reading, R.P. & Amar, A. Raptor population trends in northern Botswana: a re-survey of road transects after 20 years. *Biol. Conserv.* **224**, 87–99 (2018).
5. Thiollay, J.M. Long-term changes of raptor populations in Northern Cameroon. *J. Raptor Res.* **35**, 173–186 (2001).
6. Brooks, M.E. et al. glmmTMB balances speed and flexibility among packages for zero-inflated Generalized Linear Mixed Modeling. *The R Journal* **9,** 378–400 (2017).
7. R Core Team. *R: a language and environment for statistical computing. URL.* R Foundation for Statistical Computing, Vienna, Austria. https://www.R-project.org/ (2018).
8. Linden, A. & Mantyniemi, S. Using the negative binomial distribution to model overdispersion in ecological count data. *Ecology* **92**, 1414–1421 (2011).
9. McDonald, T.L., Erickson, W.P. & McDonald, L.L. Analysis of Count Data from Before-after Control-Impact Studies. *J. Agri., Biol. & Env. Statistics* **5**, 262-279 (2000).
10. Hedley, S.L. & Buckland, S.T. Spatial models for line transect sampling. *J. Agri., Biol. & Env. Statistics* **9**, 181 (2004).
11. Hartig, F. *DHARMa: residual diagnostics for hierarchical (multi-level/mixed) regression models.* https://cran.r-project.org/web/packages/DHARMa/vignettes /DHARMa.html (2020).
12. Fishpool, L.D.C. & Evans, M.I. (eds.) *Important bird areas in Africa and associated islands: Priority sites for conservation.* Newbury & Cambridge, UK. Pisces Publications and BirdLife International. (2001).
13. Allan, D.G., Harrison, J.A., Herremans, M., Navarro, R.A. & Underhill, L.G. Southern African geography: its relevance to birds. In: *The atlas of southern African birds. Vol. 1: Non-passerines*. Harrison, J.A., et al. (eds.). BirdLife South Africa, Johannesburg. (1997). https://www.researchgate.net/publication/310607356_Southern_African_geography_its_relevance_to_birds
14. Brooks, M. et al. The African Bird Atlas Project: a description of the project and BirdMap data-collection protocol. *Ostrich*. DOI: 10.2989/00306525.2022.2125097 (2022).
15. United Nations. *World Population Prospects 2022, Online Edition*. https://population.un.org/wpp/ (accessed 30 January 2023).
16. FAOSTAT. *Livestock Patterns*. https://www.fao.org/faostat/en/#data/EK. (accessed 24 March 2023).
17. FAOSTAT. *Land use*. https://www.fao.org/faostat/en/#data/RL. (accessed 24 March 2023).
18. Brink, A.B. & Eva, H.D. Monitoring 25 years of land cover change dynamics in Africa: a sample based remote sensing approach. *Appl. Geogr.* **29**, 501–512. (2009).
19. Roques, K.G., O'Connor, T.G. & Watkinson, A.R. Dynamics of shrub encroachment in an African savanna: relative influences of fire, herbivory, rainfall and density. *J. Appl. Ecol.* **38**, 268-280 (2001).
20. Stevens, N., Erasmus, B.F.N., Archibald, S. & Bond, W.J. Woody encroachment over 70 years in South African savannahs: overgrazing, global change or extinction aftershock? *Philos. T. R. Soc. B Biol. Sci.* **371**(1703), 20150437. (2016).
21. Venter, Z.S., Cramer, M.D. & Hawkins, H.-J. Drivers of woody plant encroachment over Africa. *Nature Comm.* **9**:2272. DOI: 10.1038/s41467-018-04616-8. (2018).
22. Smeenk, C. Comparative-ecological studies of some African birds of prey. *Ardea* **62**, 1–97 (1974).
23. Virani, M.Z., Kendall, C., Njoroge, P. & Thomsett, S. Major declines in the abundance of vultures and other scavenging raptors in and around the Masai Mara ecosystem, Kenya. *Biol. Conserv.* **144**, 746-752 (2011).
24. Brown, L. *African birds of prey.* Collins, London. (1970).
25. del Hoyo, J., Elliott, A., Sargatal, J., Christie, D.A. & de Juana, E. *Handbook of the Birds of the World Alive*. Lynx Edicions, Barcelona. Accessed June 2019. http://www.hbw.com/ (2019).

26. Bird, J.P. et al. Generation lengths of the world's birds and their implications for extinction risk. *Conserv. Biol.* **34**, 1252–1261 (2020).

27. UNEP-WCMC and IUCN. *Protected Planet: The World Database on Protected Areas (WDPA) and World Database on Other Effective Area-based Conservation Measures (WD-OECM)*, Cambridge, UK: UNEP-WCMC and IUCN. www.protectedplanet.net (2021).
