# Supplementary material for: African savanna raptors show evidence of widespread population collapse and a growing dependence on protected areas
Source: Nat Ecol Evol. 2024 Jan 4;8(1):45–56. doi: 10.1038/s41559-023-02236-0 (PMC10781635; doi:10.1038/s41559-023-02236-0)
Supplement: Supplementary file 2 — Reporting Summary [file 41559_2023_2236_MOESM2_ESM.pdf]

nature portfolio

Corresponding author(s): Phil Shaw, Darcy Ogada

Last updated by author(s): Jul 31, 2023

Reporting Summary

Nature Portfolio wishes to improve the reproducibility of the work that we publish. This form provides structure for consistency and transparency in reporting. For further information on Nature Portfolio policies, see our Editorial Policies and the Editorial Policy Checklist.

Statistics

For all statistical analyses, confirm that the following items are present in the figure legend, table legend, main text, or Methods section.

n/a	Confirmed	
☐	☒	The exact sample size (*n*) for each experimental group/condition, given as a discrete number and unit of measurement
☐	☒	A statement on whether measurements were taken from distinct samples or whether the same sample was measured repeatedly
☐	☒	The statistical test(s) used AND whether they are one- or two-sided<br>*Only common tests should be described solely by name; describe more complex techniques in the Methods section.*
☐	☒	A description of all covariates tested
☐	☒	A description of any assumptions or corrections, such as tests of normality and adjustment for multiple comparisons
☐	☒	A full description of the statistical parameters including central tendency (e.g. means) or other basic estimates (e.g. regression coefficient) AND variation (e.g. standard deviation) or associated estimates of uncertainty (e.g. confidence intervals)
☐	☒	For null hypothesis testing, the test statistic (e.g. *F*, *t*, *r*) with confidence intervals, effect sizes, degrees of freedom and *P* value noted<br>*Give P values as exact values whenever suitable.*
☒	☐	For Bayesian analysis, information on the choice of priors and Markov chain Monte Carlo settings
☒	☐	For hierarchical and complex designs, identification of the appropriate level for tests and full reporting of outcomes
☐	☒	Estimates of effect sizes (e.g. Cohen's *d*, Pearson's *r*), indicating how they were calculated

Our web collection on statistics for biologists contains articles on many of the points above.

Software and code

Policy information about availability of computer code

Data collection	No software was used for data collection
Data analysis	Data from published sources and from the Southern African Bird Atlas Project (SABAP2: https://sabap2.birdmap.africa/species) were entered or downloaded into spreadsheets in Microsoft Excel 10. They were reformatted as necessary using Excel and R code (version x64 4.1.3). GLMs and other tests were carried out using standard R functions, as described in the Methods and Supplementary Information. Copies of the code are available in figshare, at https://doi.org/10.6084/m9.figshare.23727030

For manuscripts utilizing custom algorithms or software that are central to the research but not yet described in published literature, software must be made available to editors and reviewers. We strongly encourage code deposition in a community repository (e.g. GitHub). See the Nature Portfolio guidelines for submitting code & software for further information.

Data

Policy information about availability of data

All manuscripts must include a data availability statement. This statement should provide the following information, where applicable:

- Accession codes, unique identifiers, or web links for publicly available datasets
- A description of any restrictions on data availability
- For clinical datasets or third party data, please ensure that the statement adheres to our policy

Survey data from road transects and SABAP2 have been deposited with figshare at https://doi.org/10.6084/m9.figshare.23727030. Additional background data and summary values are provided in the Supplementary Information, Tables 1-11.

Human research participants

Policy information about studies involving human research participants and Sex and Gender in Research.

Reporting on sex and gender	Our study did not involve human research participants.
Population characteristics	*Describe the covariate-relevant population characteristics of the human research participants (e.g. age, genotypic information, past and current diagnosis and treatment categories). If you filled out the behavioural & social sciences study design questions and have nothing to add here, write "See above."*
Recruitment	*Describe how participants were recruited. Outline any potential self-selection bias or other biases that may be present and how these are likely to impact results.*
Ethics oversight	*Identify the organization(s) that approved the study protocol.*

Note that full information on the approval of the study protocol must also be provided in the manuscript.

Field-specific reporting

Please select the one below that is the best fit for your research. If you are not sure, read the appropriate sections before making your selection.

☐ Life sciences ☐ Behavioural & social sciences ☒ Ecological, evolutionary & environmental sciences

For a reference copy of the document with all sections, see nature.com/documents/nr-reporting-summary-flat.pdf

Ecological, evolutionary & environmental sciences study design

All studies must disclose on these points even when the disclosure is negative.

Study description	We examined the rates at which raptor species (including vultures) were encountered during road transect surveys conducted in six African countries, in West, Central, East and southern Africa, during two time periods: the 1970s–1990s and 2000–2021. We calculated the average annual rate of change shown by 42 species, weighted by the species' range size within the countries surveyed. We also determined the population trajectories of 30 of the 42 species within South Africa during 2008–2021, using data downloaded from the Southern African Bird Atlas Project (SABAP2). Based on these sources, we: 1. Identified species at risk of extinction, by projecting annual rates of change over three generation lengths (an IUCN Red List criterion); 2. Determined whether body mass was correlated with the rate of change in abundance; 3. Compared change rates within selected protected area (PA) types and in unprotected areas (UPAs), to determine the effectiveness of site protection; 4. Compared each species' encounter rates on the PAs assessed, with those in UPAs in each period, to determine whether species had become more, or less, dependent on protected areas since the 1970s-1990s.
Research sample	We extracted encounter rates (individuals seen per 100 km driven) for 42 raptor species, each surveyed in 1–4 published studies. These were conducted in: Burkina Faso, Niger, Mali in 1969–1973 and 2003–2004 (Thiollay 2006, Ibis 148, 240–254); N. Cameroon, in 1973 and 2000 (Thiollay 2001, J. Raptor Res. 35, 173–186); N. Botswana, in 1991–1995 and 2015–2016 (Garbett et al. 2018, Biol. Conserv. 224, 87–99); and Kenya, 1970–1977 and 2003–2020 (Ogada et al. 2022, Biol. Conserv. 266, 109361). Unpublished data from an additional transect survey in N Cameroon during 2007–2010, made by two co-authors of this study (R.B. and B.M.C.), have been added. A total of 53,209 sightings of the 42 species were recorded, over a combined road survey distance of 94,151 km.<br><br>The species sampled reflected the habitats surveyed, which ranged from grassland to wooded savanna. Fifteen species were surveyed adequately only in one study area; the remaining 27 species were surveyed adequately in multiple studies. The 42 species we included represent 40% of diurnal raptor species in Africa. They are: Sagittarius serpentarius, Elanus caeruleus, Chelictinia riocourii, Milvus migrans, Necrosyrtes monachus, Gyps africanus, Gyps rueppelli, Torgos tracheliotos, Trigonoceps occipitalis, Circaetus gallicus, Circaetus beaudouini, Circaetus pectoralis, Circaetus cinereus, Terathopius ecaudatus, Circus aeruginosus, Circus pygargus, Polyboroides typus, Melierax metabates, M. poliopterus, M. canorus, Micronisus gabar, Kaupifalco monogrammicus, Accipiter badius, Butastur rufipennis, Buteo buteo, B. augur, Aquila rapax, A. nipalensis, A. spilogaster, Hieraaetus wahlbergi, Hieraaetus pennatus, Polemaetus bellicosus, Lophaetus occipitalis, Polihierax semitorquatus, Falco naumanni, F. tinnunculus, F.

rupicoloides, F. alopex, F. ardosiaceus, F. dickinsoni, F. ruficollis, F. biarmicus.

To examine trends among raptors in South Africa, we determined the direction of change in atlas reporting rates during 2008–2021, using survey data downloaded from the Southern African Bird Atlas Project (Brooks, et al. 2022 Ostrich. DOI: 10.2989/00306525.2022.2125097). Thirty of the 42 species met our selection criteria, i.e. we selected pentads (5'x5' squares) that had been surveyed at least 20 times for periods of 2–5 hours, with a minimum of five visits each in 2008–2014 and 2015–2021, and in which the target species had been recorded at least twice during the 14-year period, as confirmation of pentad occupancy. Thirty species met these selection criteria in at least 30 pentads in South Africa (median: 352 pentads; range: 38–1523), with a median of 36,165 survey visits made per species (range: 2348–164,923).

Sampling strategy

Researchers in each road transect study surveyed tracks and roads through open and wooded savanna habitats within protected areas and in unprotected areas (UPAs). PAs were defined as in the original studies. PA categories considered by study authors to afford little or no meaningful protection for wildlife, or where the degree of protection afforded was uncertain, were treated as unprotected. In the absence of historical digital maps, contemporary PA boundaries were used for both the 'early' and 'recent' survey periods in each study. These were downloaded in 2021 from The World Database on Protected Areas (WDPA) and World Database on Other Effective Area-based Conservation Measures (WD-OECM): www.protectedplanet.net. The same routes were resurveyed c. 20-40 years later. In a small number of cases the route selected during the 'early' period had not been adequately documented, in which case the nearest equivalent route was surveyed during the 'recent' survey period.

Data collection

The methods used to count raptors were standardised within studies, but differed slightly between studies. In each case, a team of 2-4 experienced observers drove slowly (averaging c. 40 km/hr) along a selected transect, and all raptors seen were recorded. Where necessary, the survey vehicle was stopped briefly to allow observers to obtain better views or to take photographs, as an aid to identification. For further details, see: Thiollay 2001, J. Raptor Res. 35, 173–186, Thiollay 2006, Ibis 148, 240–254, Garbett et al. 2018, Biol. Conserv. 224, 87–99, Ogada et al. 2022, Biol. Conserv. 266, 109361.

Timing and spatial scale

The timing of surveys was standardised with regards to season, occurring during the (northern) winter months in Burkina Faso, Niger, Mali, N. Cameroon and Kenya (to record Palearctic migrants) and in both summer and winter months in N. Botswana. Within each study, survey months were standardised between 'early' and 'recent' surveys. All surveys were conducted during daylight hours, i.e. from c. 07:00-07:30 to 18:00-18:30. The total distances surveyed in each study were as follows. Burkina Faso, Niger, Mali: 16,724 km; N. Cameroon: 4777 km; N. Botswana: 49,576 km; Kenya: 23,074 km.

Data exclusions

To minimise chance effects when transect counts from the two survey periods were compared, we restricted our analyses to species for which at least 20 individuals had been recorded during the early period, with at least five individuals seen in PAs and at least five in UPAs. As noted above, protected area categories considered by study authors to afford little or no meaningful protection for wildlife, or where the degree of protection afforded was uncertain, were treated as unprotected. African fish eagle Haliaeetus vocifer was excluded, due to its strong association with rivers and lake shores, which are poorly covered by road-based surveys. Common and lesser kestrel Falco tinnunculus and F. naumanni in West Africa where also excluded, as they were not always separated down to species level in the study in question.

Reproducibility

We calculated rates of change in the numbers of free-ranging individuals seen during large-scale surveys which were repeated after an interval of c. 20-40 years. The changes in abundance recorded reflected the changing environment and associated pressures occurring during that period, and are therefore not reproducible. No interventions or experiments were conducted as part of this study.

Randomization

In the course of four published studies, counts were made along road transects collectively spanning 94,151 km. Given the distances covered, it was not logistically possible to randomise the order or time of day at which individual transects were surveyed. Within each study, researchers controlled for the season, survey period, weather conditions and vehicle speed. In some cases, the observers involved in the first survey were unable to participate in the second. However, observer teams in each time period were highly experienced in raptor identification, and used the same protocols.
In our analysis, raptor species were assigned to either of two size classes, based on median body mass. The latter was extracted for each species from del Hoyo et al. http://www.hbw.com/ in 2019. A threshold of between 1000 g and 1400 g has typically been used in published studies to define raptor size groups. We adopted 1300 g as our threshold value, after Ogada et al. 2022. https://doi.org/10.1016/j.biocon.2021.109361.
Species were also categorised by migratory status, as: Afrotropical Sedentary; Afrotropical Migrant; Palearctic Migrant, following Buij et al. 2013. Biol. Conserv. 166, 231–246 and Clark & Davies. 2018. African Raptors. Bloomsbury. Some species were assigned to more than one migratory category, reflecting population differences.

Blinding

For purely practical reasons, blinding was not possible during each study. For example, researchers were aware of whether the transect they were surveying at a given time lay within a protected or unprotected area.

Did the study involve field work? ☒ Yes ☐ No

Field work, collection and transport

Field conditions

The survey results analysed in this study were mainly extracted from published sources, which describe surveys conducted in Burkina Faso, Niger, Mali, Cameroon, Kenya and Botswana. Details of the field conditions in which the original data were collected are provided in: Herremans et al. 2000. Biol. Conserv. 94, 31–41; Thiollay 2001, J. Raptor Res. 35, 173–186; Thiollay 2006, Ibis 148, 240–254; Virani et al. 2011. Biol. Conserv. 144, 746–752; Garbett et al. 2018, Biol. Conserv. 224, 87–99; Ogada et al. 2022, Biol. Conserv. 266, 109361. We also included a relatively small amount of unpublished survey data collected by two of the authors of the current study. These pertain to transects surveyed in Cameroon, where the field conditions were as described in Thiollay 2001 (above).
On road transect surveys, raptors were surveyed by experienced observers, from vehicles driven on roads and tracks through

	grassland, savanna woodland and farmland in protected and unprotected areas, during the 1970s-1990s and 2000s. All surveys were conducted during daylight hours, i.e. from c. 07:00-07:30 to 18:00-18:30. Given the scale of these surveys, it was not possible to control for diurnal patterns in temperature. Periods of rainfall were avoided.<br>Details of conditions under which survey visits were made to SABAP2 pentads, and the protocols applied, are given in Brooks et al. 2022. Ostrich. DOI: 10.2989/00306525.2022.2125097.
Location	Road transects were surveyed in the following areas.<br>Burkina Faso, Mali and Niger: 11°N to 20°N and 8°W to 9°E, at elevations of 200-500 m. Transects spanned two vegetation zones: the Sahel (14–20°N; rainfall 200–600 mm during the July-September wet season) and Sudan zone (11–14°N; rainfall 650–1000 mm during the June-October wet season). Surveys were made in the dry season, in mid-December to late February. Details: Thiollay 2006, Ibis 148, 240–254.<br>Northern Cameroon: 9°10' to 11°30'N, 13°24'E to 15°14'E. Rainfall: 700-900 mm during May-October. Surveys were made in the dry season (December-March). Details: Thiollay 2001, J. Raptor Res. 35, 173–186.<br>Kenya: 3°30'S to 2°15'N, 34°53'E to 38°41'E. Rainfall: 300-1700 mm. All surveys were conducted in September–May. Details: Ogada et al. 2022, Biol. Conserv. 266, 109361.<br>Botswana: 17°47'S to 22°00'S, 19°59'E to 27°43'E. Rainfall: 250-650 mm. Surveys were conducted in both the wet season (October-April) and dry season (May-September). Details: Garbett et al. 2018, Biol. Conserv. 224, 87–99.
Access & import/export	No biological samples were collected in the course of this study, or during the published studies on which our analyses are based.
Disturbance	The studies on which this study is based were purely observational. No birds were trapped, handled, ringed, tagged or sampled. No nests were knowingly disturbed. Birds were counted from existing tracks and roads. Where necessary, the survey vehicle was stopped to enable observers to better identify (and photograph) an individual bird. Any disturbance thus caused was minimal and transitory.

Reporting for specific materials, systems and methods

We require information from authors about some types of materials, experimental systems and methods used in many studies. Here, indicate whether each material, system or method listed is relevant to your study. If you are not sure if a list item applies to your research, read the appropriate section before selecting a response.

Materials & experimental systems

n/a	Involved in the study	
☒	☐	Antibodies
☒	☐	Eukaryotic cell lines
☒	☐	Palaeontology and archaeology
☐	☒	Animals and other organisms
☒	☐	Clinical data
☒	☐	Dual use research of concern

Methods

n/a	Involved in the study	
☒	☐	ChIP-seq
☒	☐	Flow cytometry
☒	☐	MRI-based neuroimaging

Animals and other research organisms

Policy information about studies involving animals; ARRIVE guidelines recommended for reporting animal research, and Sex and Gender in Research

Laboratory animals	The study did not involve laboratory animals
Wild animals	The surveys on which this study is founded were purely observational; free-ranging birds were counted, but none was caught, handled, transported or sampled. As noted above, the species observed and included in our analyses were as follows: Sagittarius serpentarius, Elanus caeruleus, Chelictinia riocourii, Milvus migrans, Necrosyrtes monachus, Gyps africanus, Gyps rueppelli, Torgos tracheliotos, Trigonoceps occipitalis, Circaetus gallicus, Circaetus beaudouini, Circaetus pectoralis, Circaetus cinereus, Terathopius ecaudatus, Circus aeruginosus, Circus pygargus, Polyboroides typus, Melierax metabates, M. poliopterus, M. canorus, Micronisus gabar, Kaupifalco monogrammicus, Accipiter badius, Butastur rufipennis, Buteo buteo, B. augur, Aquila rapax, A. nipalensis, A. spilogaster, Hieraaetus wahlbergi, Hieraaetus pennatus, Polemaetus bellicosus, Lophaetus occipitalis, Polihierax semitorquatus, Falco naumanni, F. tinnunculus, F. rupicoloides, F. alopex, F. ardosiaceus, F. dickinsoni, F. ruficollis, F. biarmicus.
Reporting on sex	The sex of the individuals counted was either not recorded or not reported in the published findings from each study. Consequently, our analyses do not examine variation in encounter rates in relation to sex.
Field-collected samples	The study did not involve collecting samples from the field.
Ethics oversight	No ethical approval or guidance was sought for the current (desk-based) study, which combines results extracted from four published road transect studies. A relatively small amount of unpublished survey data were also included. These counts were made in N. Cameroon by two co-authors (R.B. and B.M.C.), using the same protocols as in the published Cameroon study. The approach used in all of these studies was purely observational: birds were counted from a vehicle driven along existing tracks and roads, and with the minimum of disturbance.<br>Atlas survey data (from South Africa) were downloaded from SABAP2, a (purely observational) citizen science project. For details,

see: Brooks et al. 2022. Ostrich. DOI: 10.2989/00306525.2022.2125097.

Note that full information on the approval of the study protocol must also be provided in the manuscript.
